# Macrolactone Nuiapolide, Isolated from a Hawaiian Marine Cyanobacterium, Exhibits Anti-Chemotactic Activity

**DOI:** 10.3390/md13106274

**Published:** 2015-10-09

**Authors:** Shogo Mori, Howard Williams, Davey Cagle, Kristopher Karanovich, F. David Horgen, Roger Smith, Coran M. H. Watanabe

**Affiliations:** 1 Department of Chemistry, Texas A&M University, College Station, TX 77843, USA; E-Mails: smori@mail.chem.tamu.edu (S.M.); williams@cbnmr.chem.tamu.edu (H.W.); 2 Department of Natural Sciences, Hawaii Pacific University, Kaneohe, HI 96744, USA; E-Mails: dcagle@my.hpu.edu (D.C.); kkaranovich@gmail.com (K.K.); 3 Department of Veterinary Pathology, Texas A&M University, College Station, TX 77843, USA; E-Mail: ROSmith@cvm.tamu.edu

**Keywords:** macrolactone, polyketide, cyanobacteria, chemotaxis, cell cycle

## Abstract

A new bioactive macrolactone, nuiapolide (**1**) was identified from a marine cyanobacterium collected off the coast of Niihau, near Lehua Rock. The natural product exhibits anti-chemotactic activity at concentrations as low as 1.3 μM against Jurkat cells, cancerous T lymphocytes, and induces a G2/M phase cell cycle shift. Structural characterization of the natural product revealed the compound to be a 40-membered macrolactone with nine hydroxyl functional groups and a rare *tert*-butyl carbinol residue.

## 1. Introduction

Natural product extracts have long been implemented as therapeutics all over the world, such as Mesopotamia, Egypt, India, and China [1,2,3,4]. The history of herbal drug use clearly shows the significance of natural products as medicines. The natural product’s industry entered into a new era after the discovery of penicillin in 1928, which was the first pure antibiotic isolated from the fungus *Penicillium rubens* [5,6]. Although it took more than ten years for penicillin to be marketed, the remarkable success spurred researchers to perform large-scale exploration of other microorganisms. Today, this effort continues with at least 50% of all new approved drugs from 1981 to 2010 being a natural product or natural product derivative [7]. In recent years, the search for new bioactive compounds has also been hastened by the development of high throughput screening (HTS) efforts, which can assess more than 250,000 samples per day [8].

The ocean covers more than 70% of the Earth’s surface, teeming with bioactive natural products to be had from diverse sources as microorganisms, invertebrates, plants, and animals. While natural product drug therapeutics have classically come from terrestrial plants and microbes, in the last decade, a variety of marine natural products and/or their derivatives have been marketed such as ziconotide (Prialt^®^, Elan Pharmaceuticals, Dublin, Ireland) from a tropical cone snail for the treatment of pain, cytarabine (Cystosar-U, Teva Pharmaceuticals, Petah Tikya, Israel) and ecteinascidin 743 (Yondelis^®^, Pharma Mar S.A., Madrid, Spain) for the treatment of cancer, and omega-2-acid ethyl esters (Lovza^®^, GlaxoSmithKline, London, UK) for the treatment of hypertriglyceridemia [9,10,11].

Here, we evaluate marine microbial extracts for their ability to inhibit cellular chemotaxis. Cancer cell metastasis is enhanced by chemotaxis, the migration of a cell in response to a chemical stimulus [12]. Chemotaxis is observed in almost all organisms, including single-cell and multicellular organisms. Single-cell organisms, such as bacteria, approach nutritional chemicals but flee from toxic chemicals [13]. Multicellular organisms utilize the phenomenon for single cell functions, such as the movement of sperm toward an egg or of immune cells toward their targets [14,15]. The process, however, also inevitably enables some cancer cells to migrate from one location to another, triggering the development of cancers at the new site. Because many cancers are not visible and do not present symptoms until they grow sufficiently large, they may hide in other parts of the body for months or years even though the original cancer has been eliminated. Therefore, the identification of a chemical with the ability to block chemotaxis of cancer cells could provide an effective strategy for cancer treatment as an anti-metastatic agent [16]. Alternatively, compounds with anti-chemotactic behavior, e.g., FTY720 (fingolimod) can also play a key role in modulating the immune system, thus serving as effective immunosuppressive agents [17,18].

## 2. Results and Discussion

### 2.1. Evaluation of Hawaiian Marine Natural Product Extracts for Anti-Chemotactic Behavior

To identify marine natural products with potential anti-metastatic or immunosuppressive activity, microorganisms from the Hawaiian waters were collected and assayed. A total of 560 crude extract samples were evaluated for their ability to inhibit the chemotactic activity of Jurkat cells in a Boyden chamber assay [19]. Jurkats are a human cancerous T lymphocyte cell line that is capable of exhibiting migration toward a nutrient source, fetal bovine serum (FBS) [20]. A Boyden chamber is comprised of two wells separated by a membrane. The T-cells are added to the top well with fresh culture medium; a nutrient source (the chemotactic agent) and the test substance are added to the bottom well. Following incubation, the bottom well is analyzed for the presence of cells. Cells will be present in the bottom chamber if the test substance does not inhibit the chemotactic activity, but cells will remain in the top chamber if the test substance is active. FTY720, a derivative of the natural product myriocin and an approved drug for the treatment of multiple sclerosis (MS), was utilized as a control substance. *In vivo*, FTY720 is phosphorylated to form FTY720-phosphate, which resembles sphingosine 1-phosphate (S1P). S1P receptors are expressed on a wide range of cells that play a key role in the immune system and the central nervous system (CNS) [17,18]. At higher dosings, FTY720 is also cytotoxic. It has demonstrated preclinical antitumor efficacy in several cancer models [21] and has been shown to target TRPM7, a receptor linked to metastasis in breast cancer [22].

**Figure 1:**
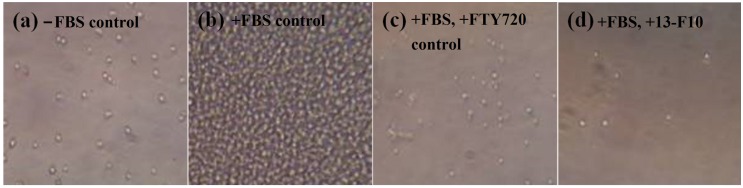
Evaluation of Jurkat cultures in the Boyden chamber assay. Images of microscope fields of the bottom well after 24 h of incubation. (**a**) Bottom well received no FBS and no test substance; (**b**) Bottom well received 10% FBS and no test substance; (**c**) Bottom well received 10% FBS and FTY720 (5 µg/mL); (**d**) Bottom well received 10% FBS and 13-F10 extract (25 µg/mL). Shrinkage of cells in (**c**) is indicative of cell death.

The crude extract samples were assayed at 25 μg/mL, consistent with typical screening concentrations for natural product extracts. Following incubation, the cells were visually observed with an inverted microscope (Figure 1). Panel (a) represents cells exposed to medium alone (no chemotactic agent) and a minimal number of cells were observed in the bottom chamber. When the chemotactic agent fetal bovine serum (FBS) was present, Panel (b), a dense lawn of cells was observed. Few cells (a combination of live and dead cells) were also observed when cells were exposed to 5 μg/mL FTY720, Panel (c). Panel (d) shows cells exposed to sample extract 13-F10, one of 50 samples that gave a positive result in this assay; the well showed the presence of a few live cells.

### 2.2. Cytotoxicity Assay for Active Crude Extracts

**Figure 2:**
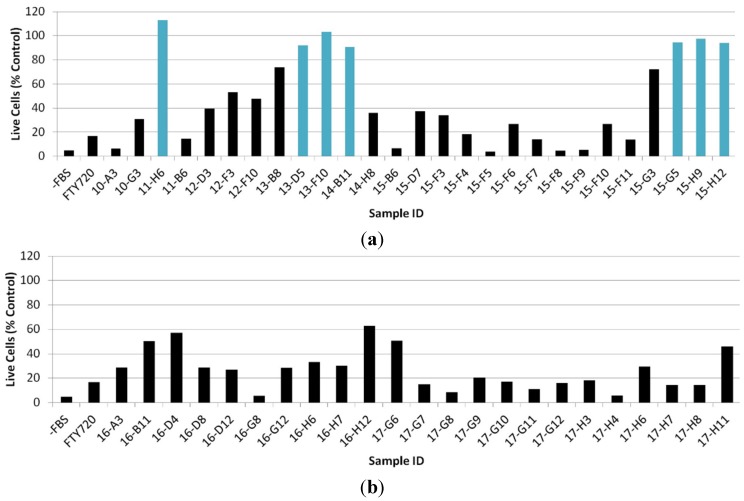
Cytotoxicity profile analysis of extracts: The turquoise bar represents samples where the percentage of live cells is ≥90% of the control. The black bar represents samples where the percentage of live cells is <90%. Controls included wells grown without FBS (−FBS) and with FTY720 (1 or 5 µg/mL). Sample codes are library plate number and coordinate on the plate. (**a**) Sample set 10- to 15- and (**b**) sample set 16- and 17-.

An ideal natural product with anti-metastatic potential would be one that inhibits the chemotactic activity but is not cytotoxic, as most chemicals that are cytotoxic to cancer cells are also cytotoxic to healthy cells. At the same time, cell toxicity alone could explain the observed lack of cell motility. Thus, the 50 samples that were shown to inhibit Jurkat cell chemotaxis were subsequently assessed for cellular cytotoxicity. After incubation with test substances, the number of live cells was analyzed by microscopy and with a CyQUANT^®^ Cell Proliferation Assay Kit. It was shown that seven out of the 50 samples did not demonstrate significant cytotoxicity against Jurkat cells at a concentration of 25 μg/mL (Figure 2) as compared to the control (T-cells in the absence of any test substances). Cytotoxicity assays were performed at 25 μg/mL as this is the highest dose in which chemotaxis assays were performed; compounds of interest would not reveal toxicity at this high concentration.

### 2.3. Evaluation of Fractionated Extracts for Anti-Chemotactic Behavior

Sample well 13-F10, an extract from a cyanobacterium whose partial 16S gene sequence showed a highly significant hit to *Okeania plumata*, was selected for further evaluation because it showed chemotaxis inhibition activity at lower dosage (10 μg/mL), and it did not show cytotoxicity at these concentrations (Figure S1). The active sample was extracted with dichloromethane or methanol and each extract was fractionated (1 min/fraction) into 24 wells in a 96-well plate. The resulting fraction plates (FP1 and FP2, respectively) were examined in the Boyden chamber assay for anti-chemotactic behavior.

**Figure 3:**
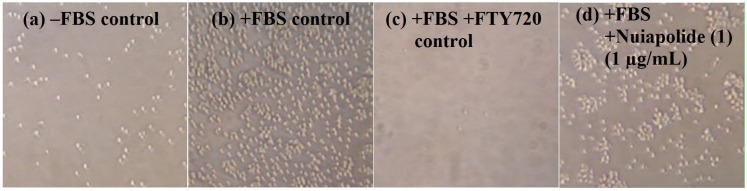
Evaluation of nuiapolide (**1**) activity at 1 µg/mL. Images of microscope fields of the bottom well after 24 h of incubation. (**a**) Bottom well received no FBS and no test substance; (**b**) Bottom well received 10% FBS and no test substance; (**c**) Bottom well received 10% FBS and FTY720 (5 µg/mL); (**d**) Bottom well received 10% FBS and nuiapolide (**1**) (1 µg/mL).

A major active component with a mass of 788.6 ± 0.2 Da (*m*/*z* 789.6, compound **1**) was identified. For additional details on the isolation/fractionation procedure, please see the Supplementary Information section and Figures S2–S6. Compound **1** was isolated to purity, its anti-chemotactic activity confirmed, and was obtained in sufficient quantities for further analysis (Figures S7 and S8). Nuiapolide (**1**) showed chemotactic activity at concentrations as low as 1 μg/mL (1.3 μM), Figure 3.

### 2.4. Cell Cycle Analysis of Nuiapolide (**1**)

Jurkat cell cultures treated with nuiapolide (**1**) were analyzed by flow cytometry to assay for the effects on the cell cycle. The population ratio of cells in each phase of the cell cycle was calculated from the histogram including: Gap 0 (G0)/Gap 1 (G1), Synthesis (S), and Gap 2 (G2)/Mitosis (M) [23,24].

**Figure 4:**
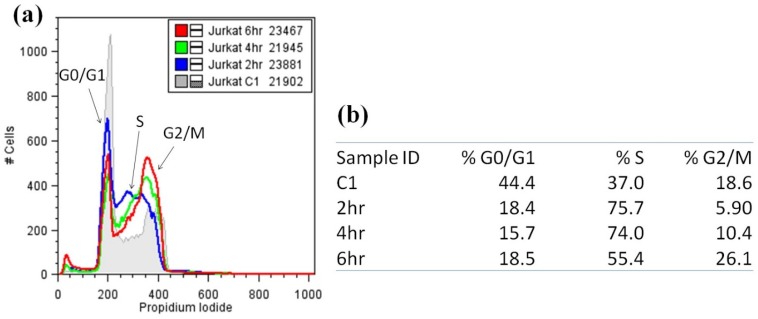
Cell cycle analysis of Jurkat cells treated with nuiapolide (**1**), 5 μg/mL. (**a**) Histograms of Jurkat cells stained with propidium iodide (numbers in the legend indicate the number of cells plotted for each treatment); (**b**) Table providing the % of cells distributed in each stage of the cell cycle.

Flow cytometry of Jurkat cells exposed to nuiapolide (**1**) showed a decrease in the population of cells in G0/G1 phase (at all the incubation times) in comparison to the control (0 h). In contrast, S phase cell populations increased with shorter exposure times (2–4 h) with cells progressing in G2/M with longer exposure to the agent (6 h, Figure 4 and Figure S9). We did not observe any 8N cells; the cell cycle is perturbed where there is a slowing or block in G2/M.

### 2.5. Structural Analysis of Nuiapolide (**1**)

**Figure 5:**
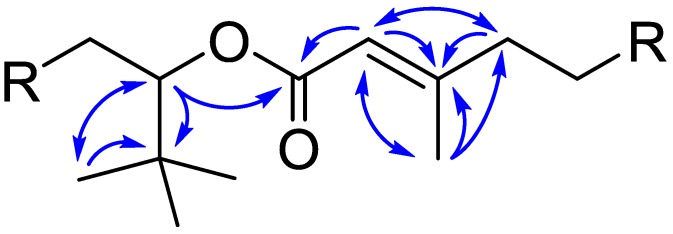
Partial structure of nuiapolide (**1**) with key HMBC correlations.

**Figure 6:**
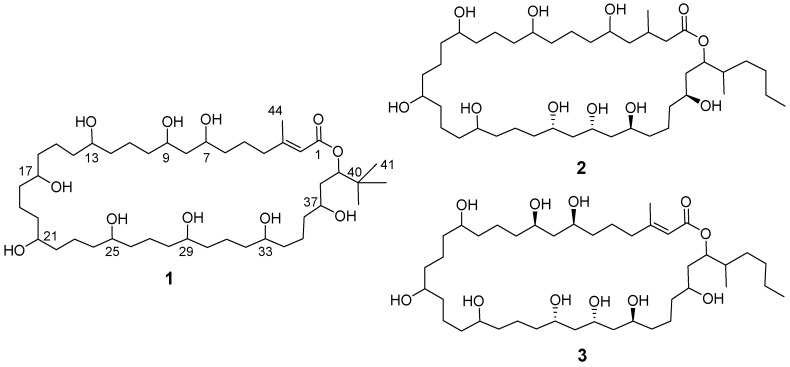
Structure of nuiapolide (**1**) and caylobolides A (**2**) and B (**3**).

The molecular formula of nuiapolide (**1**) was established as C_44_H_84_O_11_ based on HRMS (*m*/*z* [M + H]^+^ 789.6076, calcd for C_44_H_85_O_11_, 789.6092; [M + Na]^+^ 811.5895, calcd for C_44_H_84_O_11_Na, 811.5911) and requires three degrees of unsaturation. The structure was elucidated by ^1^H NMR, ^13^C NMR, ^13^C NMR DEPT, COSY, TOCSY, HSQC, HSQC-TOCSY, in methanol-*d*_4_ and DMSO-*d*_6_ (Figure 6, Table 1, and SI NMR spectra). ^13^C NMR showed 15 distinct signals, some of which (δ_C_ 20.8–21.5, 36.2–37.4, and 69.8–70.8) were overlapped with multiple peaks. HSQC correlation paired the ^1^H and ^13^C NMR signals. C-40 at δ_C_ 33.91, C-1 at δ_C_ 166.90, and C-3 at δ_C_ 161.20, which were absent in the HSQC correlation and DEPT, were predicted as R_4_C for C-40 and a substituted alkene or carbonyl carbon for C-1 and C-3. In HMBC, C-40 correlated with H-41-43 (δ_H_ 0.93) and H-39 (δ_H_ 5.02). As nine protons were predicted to be at H-41-43, C-40 and C-41-43 are due to a *tert*-butyl group attached to C-39. Based on the chemical shift and HMBC correlation between H-39 and C-1, C-39 and C-1 were assigned as an α-carbon on an oxygen linkage and carbonyl carbon, respectively, indicating that the molecule is an ester. C-1 had an HMBC correlation with alkene proton H-2. H-2 correlated with C-3, which also exhibited correlations with H-4 and H-44. Since H-44 consisted of 3 protons, this gives methyl substitution on the alkene C-3 on which C-4 extends the carbon chain further. No additional unsaturated carbons or substitutions were found except for hydroxyl groups present in the molecule, thus confirming that nuiapolide (**1**) is a macrolactone with an alkene and two side chains located near the ester functional group (Figure 5) and nine hydroxyl groups distributed throughout the saturated hydrocarbon ring structure.

Since three distinct signals of protons on hydroxyl carbons were found in the ^1^H NMR, this signified three different environments for these hydroxyl groups. These protons (locations) were indicated as H-a, H-b, and H-c at δ_H_ 3.78, 3.57, and 3.42, respectively. H-a contains two protons that had TOCSY correlations with H-4 and H-b, but they neither had nor shared COSY correlation. This suggested that there were at least two carbons between H-a and others. In HSQC-TOCSY, in addition to correlations with carbons at δ_C_ 36.2–37.4, there was a correlation with C-8 (δ_C_ 43.51), which was proposed as the carbon between each H-a. H-c consists of one proton correlated with H-39 and H-b in TOCSY but not in COSY. H-c and H-39, however, shared a correlation in COSY. These indicated that there is one carbon between H-c and H-39 and at least two carbons between H-c and H-b. Six protons in H-b were almost identical, thus their positions were likely equally distributed and spread sufficiently not to affect each other. Two distinct HSQC-TOCSY correlations with δ_C_ 36.2–37.4 and 20.8–21.5 indicated there were two isolated environments between each H-b. This confirmed that the hydroxyl groups at H-b appear at every four carbons. Because of the ring size, there cannot be more than four carbons between H-b and H-a or H-c, indicating there are three carbons between these.

**Table 1:** NMR spectral data for nuiapolide (**1**) ^a^.

Position	δ_H_ (*J* in Hz) in MeOH-*d*_4_	δ_H_ (*J* in Hz) in DMSO-*d*_6_	δ_C_ in MeOH-*d*_4_, Type	δ_C_ in DMSO-*d*_6_, Type	TOCSY	HSQC-TOCSY ^b^	HMBC ^c^
1	-	-	166.90, C	166.04, C			
2	5.74, s	5.67, s	115.73, CH	116.69, CH	4, 44, 41–43	44	1, 3, 4, 44
3	-	-	161.20, C	160.04, C			
4	2.58, m and 2.83, m	2.59, m	32.69, CH_2_	33.11, CH_2_	2, Ha, 5, 6, HC ^f^	5	2, 3, 5
5	1.60, m	1.49, m	23.86, CH_2_	24.08, CH_2_	4, H-a, a-OH, HC ^f^		4, C-i, C-iii
6 (C-i) ^d^	1.49, m	1.31, m	36.2–37.4, CH_2_	37.2–38.1, CH_2_	4, H-a, a-OH, HC ^f^		C-i, C-iii
7 (H-a) ^e^ (C-iii) ^d^	3.78, s	3.58, s	69.8–70.8, CH	69.2–70.2, CH	4, a-OH, H-b, HC ^f^	C-i, 8	
(7)-OH	-	4.44, d (4.4) or 4.47, d (4.2)	-	-	H-a, HC ^f^		8, C-i, C-iii
8	1.56, m	1.37	43.51, CH_2_	44.67, CH_2_	H-a, a-OH, HC ^f^	C-i, C-ii, C-iii	C-iii
9 (H-a) ^e^ (C-iii) ^d^	3.78, s	3.58	69.8–70.8, CH	69.2–70.2, CH	4, a-OH, H-b, HC ^f^	C-i, 8	
(9)-OH	-	4.44, d (4.4) or 4.47, d (4.2)	-	-	H-a, HC ^f^		8, C-i, C-iii
10 (C-i) ^d^	1.48–1.59, m	1.24–1.41, m	36.2–37.4, CH_2_	37.2–38.1, CH_2_	H-a, HC ^f^	C-i, C-ii, C-iii	C-i, C-ii, C-iii
11 (C-ii) ^d^	1.31–1.55, m	1.19–1.37, m	20.8–21.5, CH_2_	21.4–22.0, CH_2_	H-b, HC ^f^	C-i, C-ii, C-iii	C-i, C-iii
12 (C-i) ^d^	1.48–1.59, m	1.24–1.41, m	36.2–37.4, CH_2_	37.2–38.1, CH_2_	H-b, HC ^f^	C-i, C-ii, C-iii	C-i, C-ii, C-iii
13 (H-b) ^e^ (C-iii) ^d^	3.57, s	3.37, s	69.8–70.8, CH	69.2–70.2, CH	H-a, H-c, HC ^f^	C-i, C-ii	
(13)-OH	-	4.14, d (4.1)	-	-	H-b, HC ^f^		C-i, C-iii
14 (C-i) ^c^	1.48–1.59, m	1.24–1.41, m	36.2–37.4, CH_2_	37.2–38.1, CH_2_	H-b, HC ^f^	C-i, C-ii, C-iii	C-i, C-ii, C-iii
15 (C-ii) ^d^	1.31–1.55, m	1.19–1.37, m	20.8–21.5, CH_2_	21.4–22.0, CH_2_	H-b, HC ^f^	C-i, C-ii, C-iii	C-i, C-iii
16 (C-i) ^d^	1.48–1.59, m	1.24–1.41, m	36.2–37.4, CH_2_	37.2–38.1, CH_2_	H-b, HC ^f^	C-i, C-ii, C-iii	C-i, C-ii, C-iii
17 (H-b) ^e^ (C-iii) ^d^	3.57, s	3.37, s	69.8–70.8, CH	69.2–70.2	H-a, H-c, HC ^f^	C-i, C-ii	
(17)-OH	-	4.14, d (4.1)	-	-	H-b, HC ^f^		C-i, C-iii
18 (C-i) ^d^	1.48–1.59, m	1.24–1.41, m	36.2–37.4, CH_2_	37.2–38.1, CH_2_	H-b, HC ^f^	C-i, C-ii, C-iii	C-i, C-ii, C-iii
19 (C-ii) ^d^	1.31–1.55, m	1.19–1.37, m	20.8–21.5, CH_2_	21.4–22.0, CH_2_	H-b, HC ^f^	C-i, C-ii, C-iii	C-i, C-iii
20 (C-i) ^c^	1.48–1.59, m	1.24–1.41, m	36.2–37.4, CH_2_	37.2–38.1, CH_2_	H-b, HC ^f^	C-i, C-ii, C-iii	C-i, C-ii, C-iii
21 (H-b) ^e^ (C-iii) ^d^	3.57, s	3.37, s	69.8–70.8, CH	69.2–70.2, CH	H-a, H-c, HC ^f^	C-i, C-ii	
(21)-OH	-	4.14, d (4.1)	-	-	H-b, HC ^f^		C-i, C-ii
22 (C-i) ^d^	1.48–1.59, m	1.24–1.41, m	36.2–37.4, CH_2_	37.2–38.1, CH_2_	H-b, HC ^f^	C-i, C-ii, C-iii	C-i, C-ii, C-iii
23 (C-ii) ^d^	1.31–1.55, m	1.19–1.37, m	20.8–21.5, CH_2_	21.4–22.0, CH_2_	H-b, HC ^f^	C-i, C-ii, C-iii	C-i, C-iii
24 (C-i) ^d^	1.48–1.59, m	1.24–1.41, m	36.2–37.4, CH_2_	37.2–38.1, CH_2_	H-b, HC ^f^	C-i, C-ii, C-iii	C-i, C-ii, C-iii
25 (H-b) ^e^ (C-iii) ^d^	3.57, s	3.37, s	69.8–70.8, CH	69.2–70.2, CH	H-a, H-c, HC ^f^	C-i, C-ii	
(25)-OH	-	4.14, d (4.1)	-	-	H-b, HC ^f^		C-i, C-iii
26 (C-i) ^d^	1.48–1.59, m	1.24–1.41, m	36.2–37.4, CH_2_	37.2–38.1, CH_2_	H-b, HC ^f^	C-i, C-ii, C-iii	C-i, C-ii, C-iii
27 (C-ii)^d^	1.31–1.55, m	1.19–1.37, m	20.8–21.5, CH_2_	21.4–22.0, CH_2_	H-b, HC ^f^	C-i, C-ii, C-iii	C-i, C-iii
28 (C-i) ^d^	1.48–1.59, m	1.24–1.41, m	36.2–37.4, CH_2_	37.2–38.1, CH_2_	H-b, HC ^f^	C-i, C-ii, C-iii	C-i, C-ii, C-iii
29 (H-b) ^e^ (C-iii) ^d^	3.57, s	3.37, s	69.8–70.8, CH	69.2–70.2, CH	H-a, H-c, HC ^f^	C-i, C-ii	
(29)-OH	-	4.14, d (4.1)	-	-	H-b, HC ^f^		C-i, C-iii
30 (C-i) ^d^	1.48–1.59, m	1.24–1.41, m	36.2–37.4, CH_2_	37.2–38.1, CH_2_	H-b, HC ^f^	C-i, C-ii, C-iii	C-i, C-ii, C-iii
31 (C-ii) ^d^	1.31–1.55, m	1.19–1.37, m	20.8–21.5, CH_2_	21.4–22.0, CH_2_	H-b, HC ^f^	C-i, C-ii, C-iii	C-i, C-iii
32 (C-i) ^d^	1.48–1.59, m	1.24–1.41, m	36.2–37.4, CH_2_	37.2–38.1, CH_2_	H-b, HC ^f^	C-i, C-ii, C-iii	C-i, C-ii, C-iii
33 (H-b) ^e^ (C-iii) ^d^	3.57, s	3.37, s	69.8–70.8, CH	69.2–70.2, CH	H-a, H-c, HC ^f^	C-i, C-ii	
(33)-OH	-	4.14. d (4.1)	-	-	H-b, HC ^f^		C-i, C-iii
34 (C-i) ^d^	1.48–1.59, m	1.24–1.41, m	36.2–37.4, CH_2_	37.2–38.1, CH_2_	H-b, HC ^f^	C-i, C-ii, C-iii	C-i, C-ii, C-iii
35 (C-ii) ^d^	1.31–1.55, m	1.19–1.37, m	20.8–21.5, CH_2_	21.4–22.0, CH_2_	H-b, HC ^f^	C-i, C-ii, C-iii	C-i, C-iii
36 (C-i) ^d^	1.42, m	1.28, m	36.2–37.4, CH_2_	37.2–38.1, CH_2_	H-b, HC ^f^	37, C-i, C-ii, C-iii	C-i, C-ii, C-iii
37 (H-c) ^e^	3.42, m	3.25, m	67.58, CH	67.25, CH	H-b, c-OH, 39	C-i	
(37)-OH	-	4.15, d (5.58)	-	-	39, H-b, HC ^f^		38, C-i, C-iii
38	1.58, m	1.44, m	37.55, CH_2_	38.56, CH_2_	H-c, c-OH, HC ^f^	37, 39	C-i, C-iii
39	5.02, dd (9.7, 2.5)	4.94, dd (9.7, 2.5)	76.82, CH	76.91, CH	H-c, c-OH, 41–43, 44, HC ^f^	38	1, 37, 38, 40, 41–43
40	-	-	33.91, C	34.60, C			
41–43	0.93, s	0.85, s	25.00, CH_3_	26.27, CH_3_	2, 39		39, 40
44	1.96, s	1.87, s	23.89, CH_3_	24.91, CH_3_	39	2, 5	2, 3, 4

^a^ ^1^H NMR and ^13^C NMR in methanol-*d*_4_ and DMSO-*d*_6_ in the left four columns and 2D NMR correlations in the right four columns for each position were shown on the table; ^b^ HSQC-TOCSY correlations are ^1^H → ^13^C, and correlations at the same position are excluded; ^c^ HMBC correlations are ^1^H → ^13^C; ^d^ Three grouped ^13^C NMR peaks are marked as C-i, C-ii, and C-iii; ^e^ Three ^1^H NMR peaks of protons on hydroxyl carbons are marked as H-a, H-b, and H-c; ^f^ HC is overlapped hydrocarbon peaks at 1.31–1.59 (methanol-*d*_4_) and 1.19–1.41 (DMSO-*d*_6_) in ^1^H NMR.

The proposed location of hydroxyl groups was confirmed by the number of carbons in environments through the ring structure (Figure S10). Nine hydroxyl carbons were found at δ_C_ 67.58 (C-37) and δ_C_ 69.8–70.8 (C-7, C-9, C-13, C-17, C-21, C-25, C-29, and C-33). There were 16 α-carbons of hydroxyl groups (except for C-8) at δ_C_ 37.55 (C-38) and 36.2–37.4 (C-6, C-10, C-12, C-14, C-16, C-18, C-20, C-22, C-24, C-26, C-28, C-30, C-32, C-34, and C-36). Seven β-carbons (except for C-5) were located at δ_C_ 20.8–21.5 (C-11, C-15, C-19, C-23, C-27, C-31, and C-35). The location of hydroxyl groups was further confirmed by ^1^H NMR, ^13^C NMR, COSY, TOCSY, and HMBC in DMSO-*d*_6_, which showed signals of hydroxyl protons (Table 1 and SI NMR spectra). The new peaks appeared at δ_H_ 4.14, 4.15, 4.44, and 4.47. COSY correlations showed that the peaks at δ_H_ 4.44 and 4.47 were from hydroxyl groups at position H-a, the peak at δ_H_ 4.14 was from H-b hydroxyl groups, and the peak at δ_H_ 4.15 was from position H-c, which was confirmed by a TOCSY correlation with H-39. HMBC showed correlations between δ_H_ 4.44/4.47 and C-8, between δ_H_ 4.14 and δ_C_ 36.2–37.4/69.8–70.8, and between δ_H_ 4.15 and C-38. Based on the above analyses, the structure of nuiapolide (**1**) was determined, as shown in Figure 6.

The structure suggested that the compound belongs to the polyketide class of natural products and displays structural similarity to the caylobolides, which were shown to be cytotoxic against some cancer cell lines [25,26]. Nuiapolide (**1**) and caylobolides A (**2**) and B (**3**) share the core structure of a macro-lactone. They contain a large saturated carbohydrate ring with several hydroxyl groups and similar side chains around the ester functional group. The number of carbons in the ring structure, however, was slightly greater in compound **1** (39 carbons) than in caylobolides (35 carbons). Given the structure of **1** and its Hawaiian origins, we named it nuiapolide (**1**). Nuiapo means big circle in Hawaiian and “ide” indicates the macrolide.

## 3. Materials and Methods

### 3.1. General Experimental Procedures

High resolution ESI-MS spectra were measured on an Agilent 6530 QTOF mass spectrometer with an Agilent 1200 HPLC system. Unit-resolution ESI-MS spectra were performed on a Thermo Finnigan LCQ Deca XP Max ion trap mass spectrometer using ESI ionization interfaced with an Agilent 1100 HPLC, Thermo Finnigan Surveyor photodiode array (PDA) detector, and a SEDEX 75 Evaporative light scattering detector (ELSD). Preparative HPLC was performed using an Agilent 1100 binary preparative system with an Agilent multi-wavelength UV detector. An inverted microscope (VWR, Radnor, PA, USA) was used for evaluation of Jurkat cells. Photographs of the cells were taken with 100× magnification (10× ocular and 10× objective lens) and a camera (Olympus, Shinjuku, Tokyo, Japan) equipped with a modified lens piece, which fits over the microscope eyepiece. Cell cycle analysis of Jurkat cells was performed with a flow cytometer (FACSCalibur, BD Biosciences, San Jose, CA, USA**)**. Flow cytometric data were analyzed using ModFitLT (Verity Software House, Topsham, ME, USA) and plotted using FlowJo (FlowJo LLC, Ashland, OR, USA). NMR spectra were acquired on a Bruker Avance III 500 MHz spectrometer equipped with a 5 mm H-C-N cryoprobe (Bruker Corporation, Billerica, Massachusetts, USA) at 500 MHz for ^1^H NMR and 125 MHz for ^13^C NMR in methanol-*d*_4_ or DMSO-*d*_6_.

### 3.2. Reagents and Chemicals

Jurkat clone E6-1, its culture medium RPMI 1640, and fetal bovine serum (FBS) were purchased from American Type Culture Collection (ATCC, Manassas, VA, USA). Triton X-100, DNase-free RNase, propidium iodide, and ampules of NMR solvents (methanol-*d*_4_ and DMSO-*d*_6_) were purchased from Sigma-Aldrich (St. Louis, MO, USA).

### 3.3. Sample Preparation for Assay Analysis

The 560 crude extracts were prepared from freeze-dried samples of microorganisms collected from near-shore environments in the main Hawaiian Islands. Active sample 13-F10, was prepared from a colonial cyanobacterium (071905-NII-01). Specifically, the freeze-dried cyanobacterium sample (105 mg) was extracted in H_2_O (10 mL) with sonication. The water extracted was filtered and loaded on a C-4 solid phase extraction (SPE) cartridge (500 mg, 300 Å). The cartridge was washed with H_2_O (10 mL) and the H_2_O wash was discarded. The column was then eluted with methanol/water mixtures (33:67, 67:33, and 100:0; 6 mL each). The combined fractions were dried under vacuum (3.9 mg) and added to our extract libraries as 13-F10. Prior to assay, a small portion of the extract was dissolved in methanol/ethyl acetate/*t*-butyl methyl ether (60:30:10) (MET) and transferred to a 96-well plate. The plates were stored desiccated and vacuum packed at −20 °C. Each 96-well plate of extracts was solubilized in MET and evaluated in our chemotaxis transwell assay.

#### 3.3.1. Details on Cyanobacteria Biomass

Colonies of the red filamentous cyanobacterium were collected by hand on July 19, 2005 at a depth of 12–20 m in waters adjacent to Lehua Rock, an islet off the east shore of Niihau, Hawaii. Samples were kept on ice in the field before freezing at −15 °C. The frozen samples were then freeze-dried. A dried voucher specimen (071905-NII-01) is deposited in the Department of Natural Sciences, Hawaii Pacific University and a partial 16S rRNA gene sequence was deposited in the NCBI GenBank under the Accession No. (KT852581).

#### 3.3.2. DNA Extraction and Sequencing

DNA was extracted using a Qiagen DNeasy kit. A small portion of frozen biomass was thawed and warmed to 55 °C. Lysis buffer (“ATL” buffer, 180 μL) and proteinase K solution (600 mAU/mL; 20 μL) were added, and the mixture was vortexed, then incubated overnight a 55 °C. After warming the mixture to 70 °C, the sample was again vortexted, and chaotropic “AL” buffer (200 μL) was added, followed by a 10 min incubation. The sample was then mixed thoroughly with ethanol (200 μL) and pipetted into the DNeasy mini-spin column, which was placed in a collection tube and centrifuged at 6000 *g* for 1 min. The column was washed by centrifuging again at 6000 *g* for 1 min after adding the chaotropic elution buffer “AW1” (500 μL) and again with wash buffer “AW2” (500 μL) and centrifuging again at 20,000 *g* for 3 min. Elution buffer “AE” (100 μL) was added and incubated at room temperature for 1 min, before centrifuging at 6000 *g* for 1 min. The DNA eluate was collected and stored at −20 °C.

Primers were developed by Nubel and coworkers (1997) to amplify an approximate 700 bp region of 16S that allows the specific amplification of cyanobacterial 16S genes sequences [27]. The partial sequencing of 16S was accomplished by adding 1 μL of thawed isolated DNA to a mixture of H_2_O (26 μL), dNTP (1.0 μL, Eppendorf, Hamburg, Germany), forward primer (1.0 μL, 20 pmol/μL, CYA106F: CGGACGGGTGAGTAACGCGTGA), and reverse primers (1.0 μL, 20 pmol/μL, a 1:1 ratio of CYA781Ra: GACTACTGGGGTATCTAATCCCATT and CYA781Rb: GACTACAGGGGTATCTAATCCCTTT) in a PCR tube. The mixture was heated to 95 °C for 2 min and the following mixture was added: 14.75 μL H_2_O, 5 μL 10× reaction buffer, and 0.25 μL Taq polymerase (Eppendorf, Hamburg, Germany). The following cycles were applied: 10 cycles, 95 °C for 30 s, 65 °C for 30 s, the cool −1 °C; 30 cycles, 95 °C for 30 s, 55 °C for 30 s, 68 °C for 1 min. Finally, the PCR product was held at 68 °C for 7 min and then stored at 4 °C. The PCR product was checked for a single correctly sized product by agarose gel electrophoresis, and the band was prepared for sequencing using a Qiagen QIAquick spin kit per instructions. Sequencing was conducted by the by the Greenwood Molecular Biology Facility (University of Hawaii) employing the ABI Prism BigDye Terminator and Primer Cycle Sequencing Chemistries and an ABI 377XL capillary sequencer.

A tblastx search against the NCBI database with *E*-value cutoff of ≤1.0 × 10^−3^ revealed a highly significant hit to *Okeania plumata* (KC986934.1) [28].

#### 3.3.3. Scaled-Up Extraction of the Active Cyanobacterial Species

For process and storage efficiency, the bulk of our library samples are routinely extracted in organic solvents. Sample 071905-NII-01 (5.6 g dry wt) was extracted repeatedly with methanol (65 mL and 2 × 50 mL) followed by dichloromethane (2 × 50 mL). The methanol extracts were combined and solvent was removed under vacuum to yield 1.8 g of extract residue. The dichloromethane extracts were also combined and solvent removed under vacuum to yield 34 mg. Analytical amounts of both extracts were subject to bioassay-linked fractionation in order to identify the active component(s) of the organism.

#### 3.3.4. Bioassay-Linked Fractionation of Methanol and Dichloromethane Extracts of the Cyanobacterium 071905-NII-01

Both extracts (dichloromethane, 1.7 mg, FP1; methanol, 2.0 mg, FP2) were fractionated in two runs each by reversed-phase HPLC (Phenomenex Luna C18-2 column, 5 μm, 4.6 mm × 250 mm, Torrance, CA, USA) using a linear gradient of acetonitrile/water (FP1: 50:50 to 100:0 over 0–15 min, then 100% acetonitrile from 15–26 min; FP2: 20:80 to 100:0 over 0–15 min, then 100% acetonitrile from 15–26 min; flow rate: 1.0 mL/min; UV detection: 200–600 nm). Extract components eluting between 2.0 and 26.0 min were collected as 60-s fractions into a deep 96-well plate. For each extract, both HPLC runs were collected into the same deep-well plate and solvent was removed from the plates under vacuum in a centrifugal concentrator at room temperature. Fractions were reconstituted with MET and apportioned into low profile 96-well plates for bioassay, averaging 45 and 60 μg/well for FP1 and FP2, respectively. Prior to assay, the fractions were reconstituted and diluted in MET and tested as average concentrations of 1, 10, and 25 μg/mL. For FP1, the activity concentrated in fractions eluting at 4.0–5.0 and 12.0–14.0 min. And for FP2, activity concentrated in fractions eluting at 9.0–10.0 and 16.0–17.0 min. Taken together, LCMS of both active regions in both fraction plates indicated the presence of the same components in each of the two active regions. FP1 fractions had only very minor impurities. The major component in the earliest active region for each fraction was absent from adjacent inactive fractions and showed a molecular weight of 788.6 (ESI-positive mode *m*/*z* 789.6, MH^+^; ESI-negative mode *m/z* 787.6 [M − H]^−^).

#### 3.3.5. Isolation of Nuiapolide (**1**)

The major component from the first active fraction in FP1 and FP2 was isolated from the methanol extract, guided by LCMS (ESI-positive mode *m*/*z* 789.6, MH^+^; ion trap analyzer). The methanol extract (1020 mg) was dissolved in methanol and cleaned up for HPLC by eluting in seven batches from an SPE column (C18 silica, 1 g, EM 110167-4) with methanol (17 mL each). The eluate was concentrated under vacuum to dryness (996 mg), reconstituted in methanol/water 90:10 (14 mL) and chromatographed in seven portions by preparative reversed phase HPLC (Phenomenex Luna C18-2 column, 5 μm, 22 mm × 250 mm, Torrance, CA, USA; flow rate: 18 mL/min; methanol/water linear gradient 70:30 to 100:0 from 0–15 min, then 100% methanol from 15–30 min). Peaks were collected based on absorbance at 210 nm and analyzed by LCMS. Compound **1**, nuiapolide, eluted at 8.6 min (50.7 mg, 0.89% yield, dry wt). The purity of nuiapolide (**1**) (>95%) was determined by LCMS-ELSD analysis (Phenomenex Luna C18-2 column, 5 μm, 2 mm × 250 mm, Torrance, CA, USA; flow rate: 0.20 mL/min; acetonitrile/water gradient 50:50 to 100:0 over 0–10 min, then 100% acetonitrile 10–20 min; ELSD 50 °C; PDA 200–600 nm).

#### 3.3.6. Structural Analysis of Nuiapolide (**1**)

Nuiapolide (**1**): chartreuse amorphous solid; ${\left[\alpha \right]}_{D}^{25}$ −12 (CH_3_OH, *c* 0.341); ^1^H and ^13^C NMR data (methanol-*d*_4_ and DMSO-*d*_6_), see Table 1 and SI figures; HR-TOF-ESIMS, *m*/*z* [M + H]^+^ 789.6076 (calcd for C_44_H_85_O_11_, 789.6092, ∆ −2.2 ppm), [M + Na]^+^ 811.5895 (calcd for C_44_H_84_O_11_Na, 811.5911, ∆ −2.0 ppm). NMR spectra were recorded on a Bruker Avance III. The purified sample (500 μg) was dissolved in 500 μL of methanol-*d*_4_ or DMSO-*d*_6_, which was transferred into a 3 mm NMR sample tube (Wilmad LabGlass, Vinelandk, NJ, USA). ^1^H NMR, ^13^C NMR, ^13^C NMR DEPT, COSY, TOCSY, HSQC, HSQC-TOCSY, and DMSO-*d*_6_ were acquired in methanol-*d*_4_ at 298 K (SI figures for spectra; Table 1 for assignments), and ^1^H NMR, ^13^C NMR, COSY, TOCSY, and HMBC were recorded in DMSO-*d*_6_ at 315 K (SI figures).

### 3.4. Chemotaxis Assay with Jurkat Cells

Jurkat cells were cultivated in the cell culture medium (RPMI 1640 with 10% FBS) and maintained in 75 cm^2^ culture flasks at 37 °C in 5% CO_2_. Cells were harvested by centrifugation (10 min at 1000 rpm), washed twice with the fresh culture medium without serum, and resuspended in fresh culture medium. The number of cells was counted with a hemocytometer, and the cell culture volume adjusted to give a cell stock solution of 2,000,000 cells/mL. Transwell^®^ (24-well) plates with 5.0 μm permeable polycarbonate membrane supports (Boyden chamber, Costar, Corning, NY, USA) were used. The bottom well contained 600 μL of cell culture medium with 1, 10, or 25 μg/mL of sample or 5 μg/mL FTY720, and 100 μL of cells (200,000 cells) was added to the top well. Two controls without test samples were utilized to observe cell migration induced by FBS. In the first control, the bottom well received MET solution alone. In the second control, fresh RPMI medium without FBS was used for the bottom well in order to confirm the enhancement of Jurkat chemotaxis by FBS. After incubation for 24 h, the cells were collected from the bottom well to observe how many cells had migrated.

### 3.5. Cytotoxicity Assay with Jurkat Cells

Following 24 h incubation of Jurkat cells in Transwell^®^ plates, as detailed above, the cells were transferred to 1.5 mL eppendorf tubes and harvested by centrifugation for 15 min at 14,000 rpm. The medium was removed and the cells washed with phosphate buffered saline (PBS) without manganese and calcium ions. The resulting cell pellets were frozen in −80 °C for subsequent lysis. Cells were lysed and stained with green fluorescent (GR) dye utilizing a CyQUANT^®^ Cell Proliferation Assay Kit (Invitrogen, Life Technologies, Thermo Fisher Scientific, Waltham, MA, USA). Samples were subsequently transferred into black 96-well plates and analyzed using a 96-well fluorescence plate reader to measure relative fluorescence (FLx800 Fluorescence Reader, BioTek, Winooski, VT, USA).

### 3.6. Cell Cycle Analysis by Flow Cytometry

The effect of nuiapolide (**1**) on the cell cycle was assessed by flow cytometry. Jurkat cells were harvested by centrifugation and counted following the same procedures as detailed in previous sections. Cells were added to RPMI cell culture medium in 24-well plates to give a final concentration of 400,000 cells/mL, 500 μL total volume. Nuiapolide (**1**) was added (5.0 μg/mL) and the cells were incubated at 37 °C in 5% CO_2_ for 2, 4, or 6 h. The resulting cells were prepared for flow cytometry analysis based on published procedures [29]. Cells were transferred into a 15-mL falcon tube and harvested by centrifugation at 1000 rpm for 10 min. Cells were subsequently washed with 5 mL of PBS and pelleted at 1000 rpm for 6 min. The supernatant was removed and the cells were resuspended in 0.5 mL of PBS. The cell suspension was transferred into 4.5 mL of ice-cold ethanol and stored on ice (or −20 °C) for more than 2 h. The ethanol fixed cells were collected by centrifugation at 1000 rpm for 5 min. The cell pellet was resuspended in 5 mL of PBS, incubated for 1 min and centrifuged again at 1000 rpm for 5 min. The cell pellet was finally resuspended in 1 mL of freshly prepared staining solution (PBS, 0.1% of Triton X-100, and 0.2 mg/mL of DNase-free RNase, and 0.02 mg/mL of propidium iodide), and incubated at room temperature for 30 min before analyzing the samples by flow cytometry using a 488 nm argon laser for excitation and a 585/42 bandwidth filter for fluorescence detection.

## 4. Conclusions

Cell-based screening of marine natural product extracts has led to the discovery of nuiapolide (**1**) that inhibits chemotaxis of Jurkats and induces a predominant G2/M phase shift in the cell cycle. The large, 40-membered macrolactone contains a rare *tert*-butyl carbinol residue, which is present in cyanobacterial compounds apratoxin A, a cyclodepsipeptide with potent anti-cancer activity, and a couple other macrolide compounds, madangolide and laingolide [30,31,32]. Nuiapolide resembles a previously described class of anti-tumor natural products, the caylobolides, which were also isolated from cyanobacteria. Caylobolide A and B were isolated from *Moorea producens* (previously *Lyngbya majuscula*) and *Phormidium* spp. respectively [25,26]. During the process of publishing this work, bastimolide A, a potent anti-malarial compound was reported, as well as amantelide A and B. Both sets of compounds were reported from or suspected as *Okeania* species and represent polyhydroxylated macrolides with a *tert*-butyl carbinol group and may reflect analogs or isomers of nuiapolide (**1**) [33,34].

Given the large molecular structure of nuiapolide (**1**), further evaluation of this family of compounds as anti-metastatic or immunosuppressive agents would greatly benefit from elucidation of the biosynthetic gene cluster for bio-engineering purposes or the synthesis of a chemical library of compounds based upon a simplified structural motif.

## Supplementary Files

Supplementary File 1
