# Supplementary material for: Macrolactone Nuiapolide, Isolated from a Hawaiian Marine Cyanobacterium, Exhibits Anti-Chemotactic Activity
Source: Mar Drugs. 2015 Oct 9;13(10):6274–90. doi: 10.3390/md13106274 (PMC4626689; doi:10.3390/md13106274)
Supplement: Supplementary File 1 [file marinedrugs-13-06274-s001.docx]

**Supplementary Information**

| **Contents** | **Page Number** |
| --- | --- |
| Figure S1. Chemotaxis assay of library extract 13-F10 at lower doses | S2 |
| Figure S2. Bioassay-linked fractionation of extracts from cyanobacterium 071905-NII-01 | S2 |
| Figure S3. PDA map of active fractions in fraction plate FP1 | S4 |
| Figure S4. PDA map of active fractions in fraction plate FP2 | S4 |
| Figure S5. LCMS analysis of active fraction A3 in fraction plate FP1 | S5 |
| Figure S6. LCMS analysis of active fractions A11/12 in fraction plate FP2 | S5 |
| Figure S7. Purity of nuiapolide (**1**) and NP982 | S6 |
| Figure S8. Chemotaxis assay of FP1 active fractions | S7 |
| Figure S9. Cell cycle analysis of nuiapolide (**1**) treated Jurkat cells | S8 |
| Figure S10. Number of carbons in ^13^C NMR grouped regions | S9 |
| Figure S11. Spectral data of nuiapolide (**1**) | S9 |
| Figure S12. ^1^H NMR of nuiapolide (**1**) in methanol-*d*_4_ | S10 |
| Figure S13. ^13^C NMR of nuiapolide (**1**) in methanol-*d*_4_ | S11 |
| Figure S14. DEPT-90 of nuiapolide (**1**) in methanol-*d*_4_ | S12 |
| Figure S15. DEPT-135 nuiapolide (**1**) in methanol-*d*_4_ | S13 |
| Figure S16. COSY of nuiapolide (**1**) in methanol-*d*_4_ | S14 |
| Figure S17. TOCSY of nuiapolide (**1**) in methanol-*d*_4_ | S15 |
| Figure S18. HSQC of nuiapolide (**1**) in methanol-*d*_4_ | S16 |
| Figure S19. HSQC-TOCSY of nuiapolide (**1**) in methanol-*d*_4_ | S17 |
| Figure S20. HMBC of nuiapolide (**1**) in methanol-*d*_4_ | S18 |
| Figure S21. ^1^H NMR of nuiapolide (**1**) in DMSO-*d*_6_ | S19 |
| Figure S22. ^13^C NMR of nuiapolide (**1**) in DMSO-*d*_6_ | S20 |
| Figure S23. COSY of nuiapolide (**1**) in DMSO-*d*_6_ | S21 |
| Figure S24. TOCSY of nuiapolide (**1**) in DMSO-*d*_6_ | S22 |
| Figure S25. HSQC of nuiapolide (**1**) in DMSO-*d*_6_ | S23 |
| Figure S26. HMBC of nuiapolide (**1**) in DMSO-*d*_6_ | S24 |

Details on Evaluation of Fractionated Extracts for Anti-Chemotactic Behavior

The resulting fraction plates (FP1 and FP2, respectively) were examined in the Boyden chamber assay for anti-chemotactic behavior. Each extract provided three active fractions including: A3 (4–5 min), A11 (12–13 min), and A12 (13–14 min) from FP1, and A8 (9–10 min), B2 (15–16 min), and B3
(16–17 min) from FP2 (Figure S2).

The LC profiles and activity patterns of FP1 and FP2 showed similar patterns except the retention times of the active components were shifted reflecting the different gradients of the separation. This suggested the same active components were present in both samples with FP1 wells A3 and A11/A12 corresponding to FP2 wells A8 and B2/B3, respectively and likewise, activity at A11 and A12 from FP1 (corresponding to B2 and B3 from FP2). This was confirmed by photodiode array (PDA) map and LC/MS (Figures S3–S6). Wells FP1-A3/FP2-A8 showed a major active component with a mass of 788.6 ± 0.2 Da (*m*/*z* 789.6, compound **1**). Compound **1** was subjected to purification by LC-MS.


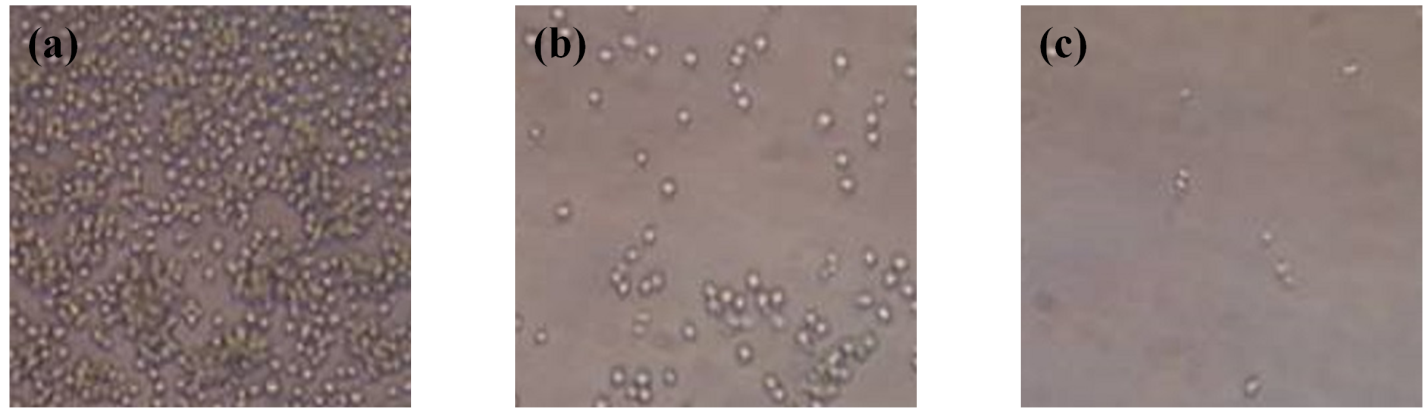

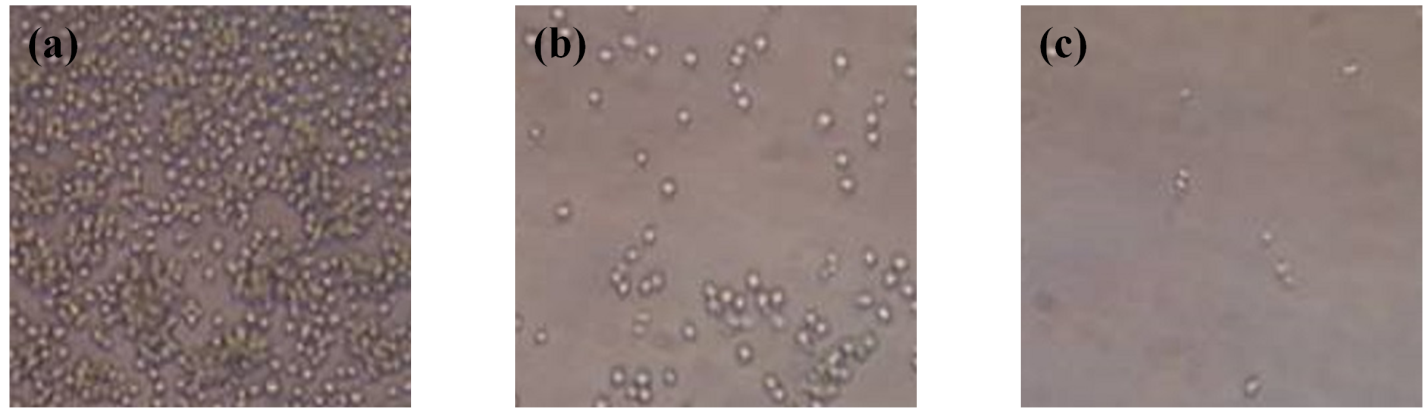

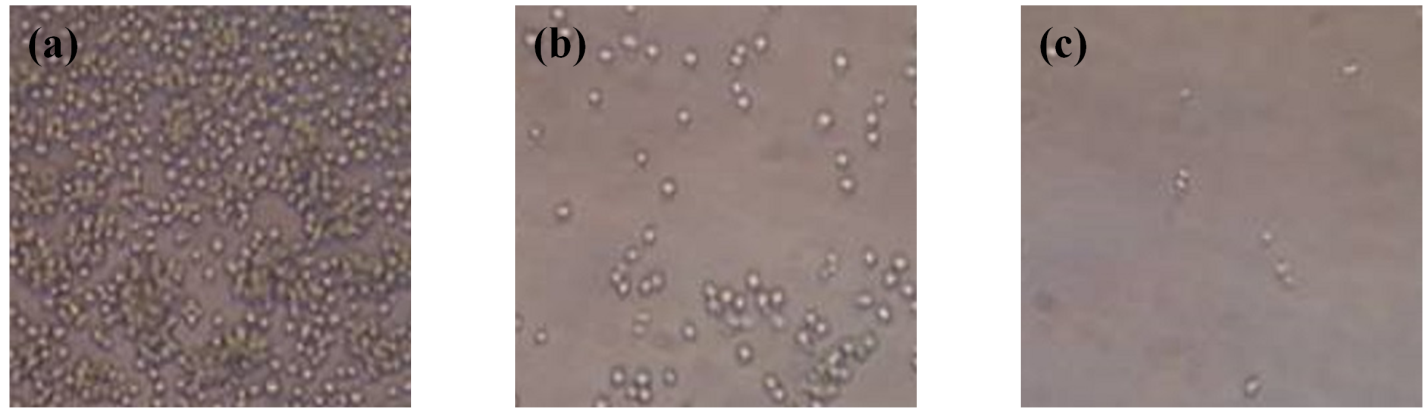


**Figure S1.** Chemotaxis assay of library extract 13-F10 at lower doses. (**a**): 1 µg/mL;
(**b**): 10 µg/mL; (**c**): 25 µg/mL.

| 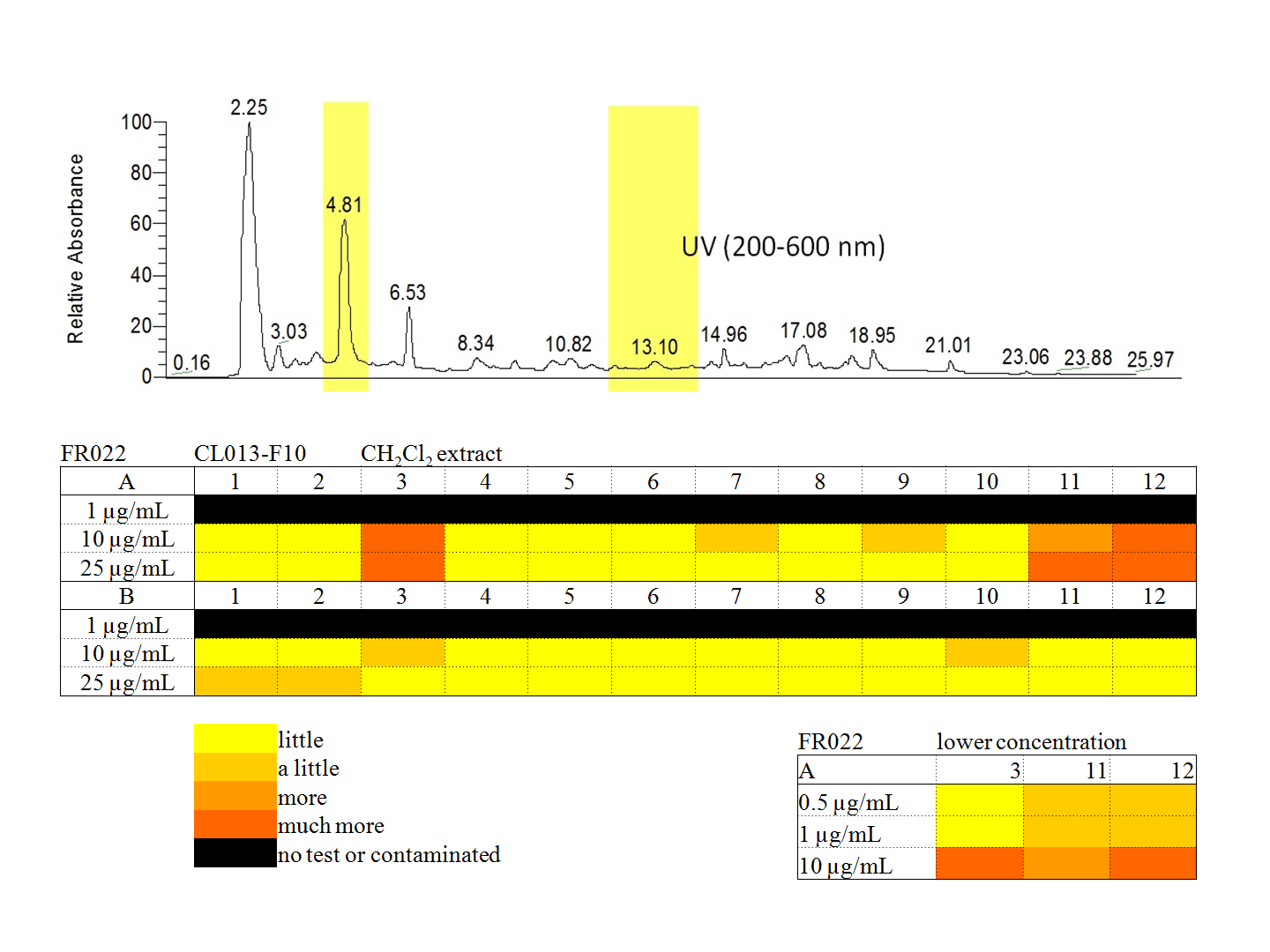 |
| --- |
| (**a**) |

**Figure S2.** *Cont.*

| 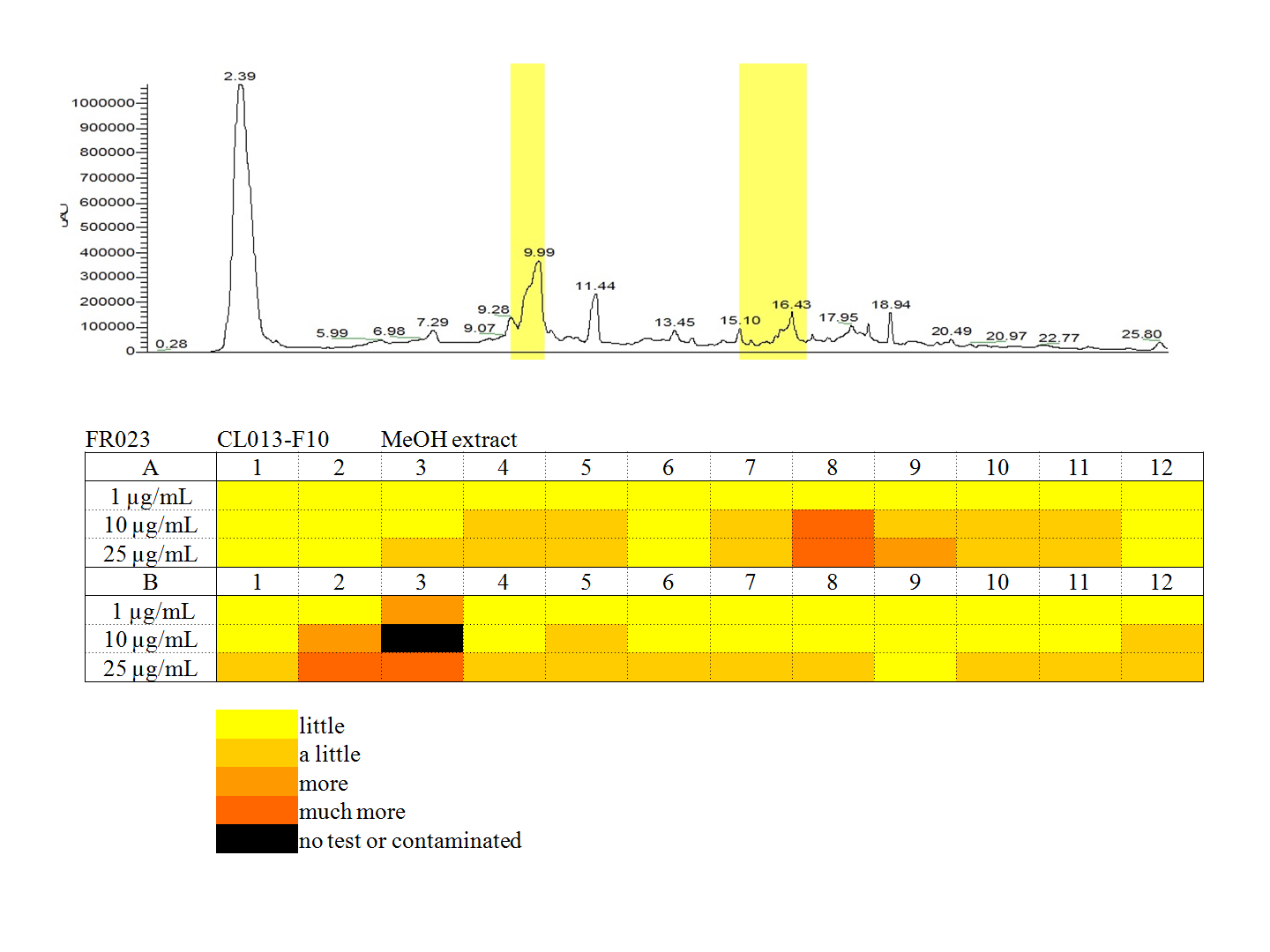 |
| --- |
| (**b**) |

**Figure S2.** Bioassay-linked fractionation of extracts from cyanobacterium 071905-NII-01. The cyanobacterial biomass was extracted with methanol followed by dichloromethane. The extracts were fractionated by reverse-phase HPLC (see experimental for conditions) into 96-well plates (1 min/well) and each resulting fraction plate—(**a**) dichloromethane (FP1) and (**b**) methanol (FP2)—was assessed for inhibition of Jurkat chemotactic activity. The yellow bars in the HPLC profile identify the active fractions: (**a**): 4–5, 12–13, and
13–14 min; (**b**): 9–10, 15–16, and 16–17 min. Yellow to orange colors (low to high inhibition) in the table represents the level of activity.


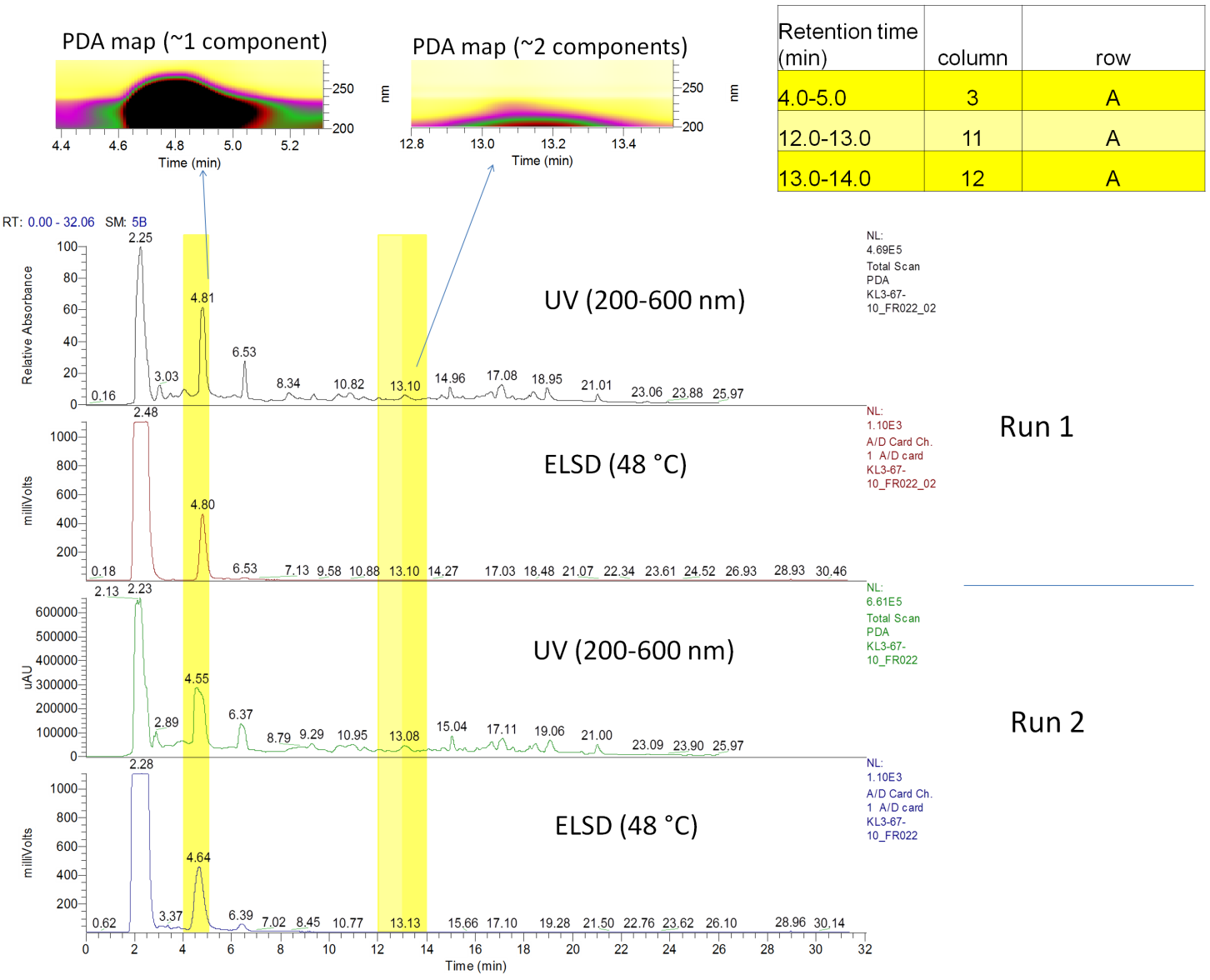


**Figure S3.** PDA map of active fractions in fraction plate FP1, the dichloromethane extract of 071905-NII-01.


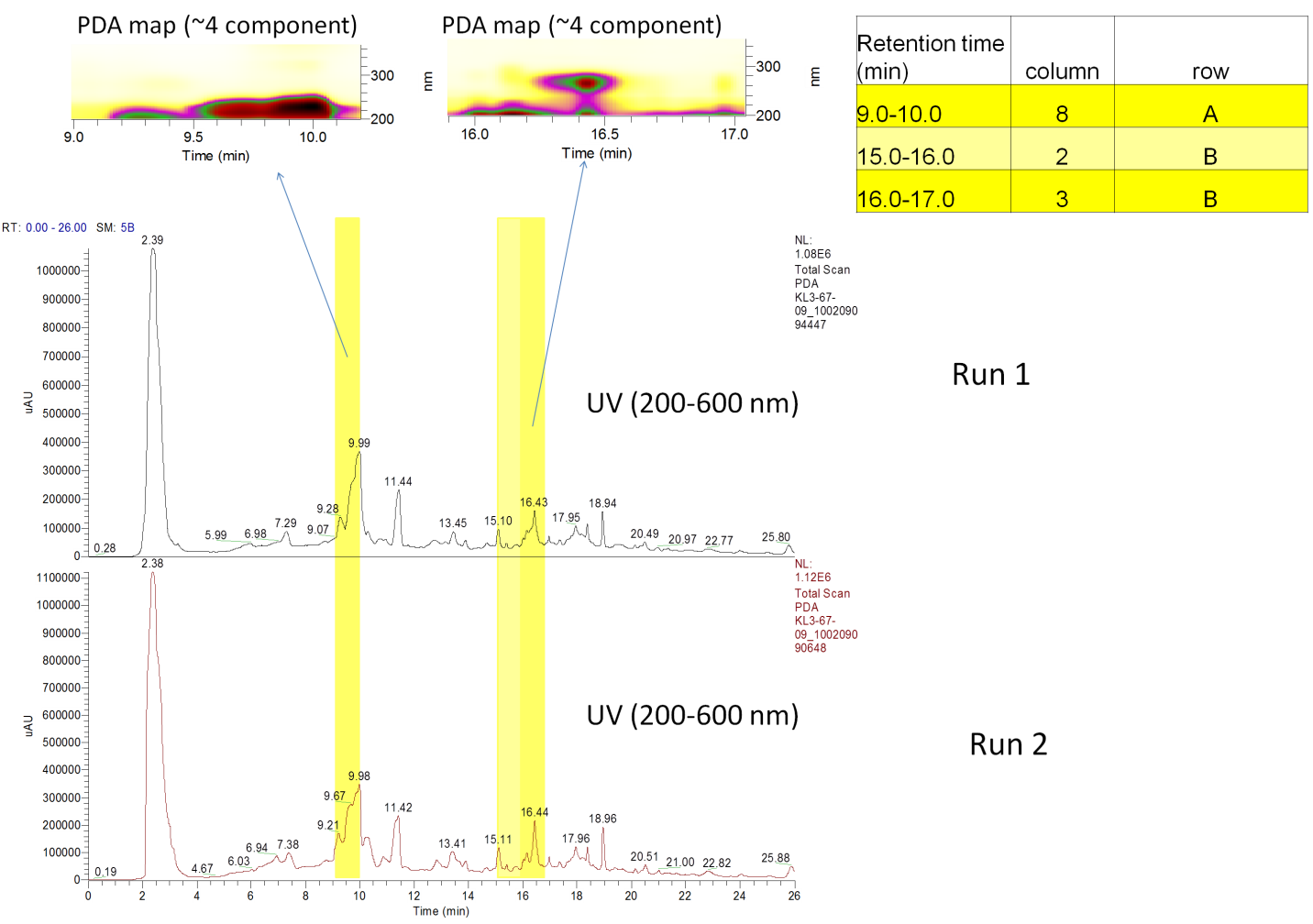


**Figure S4.** PDA map of active fractions in FP2.


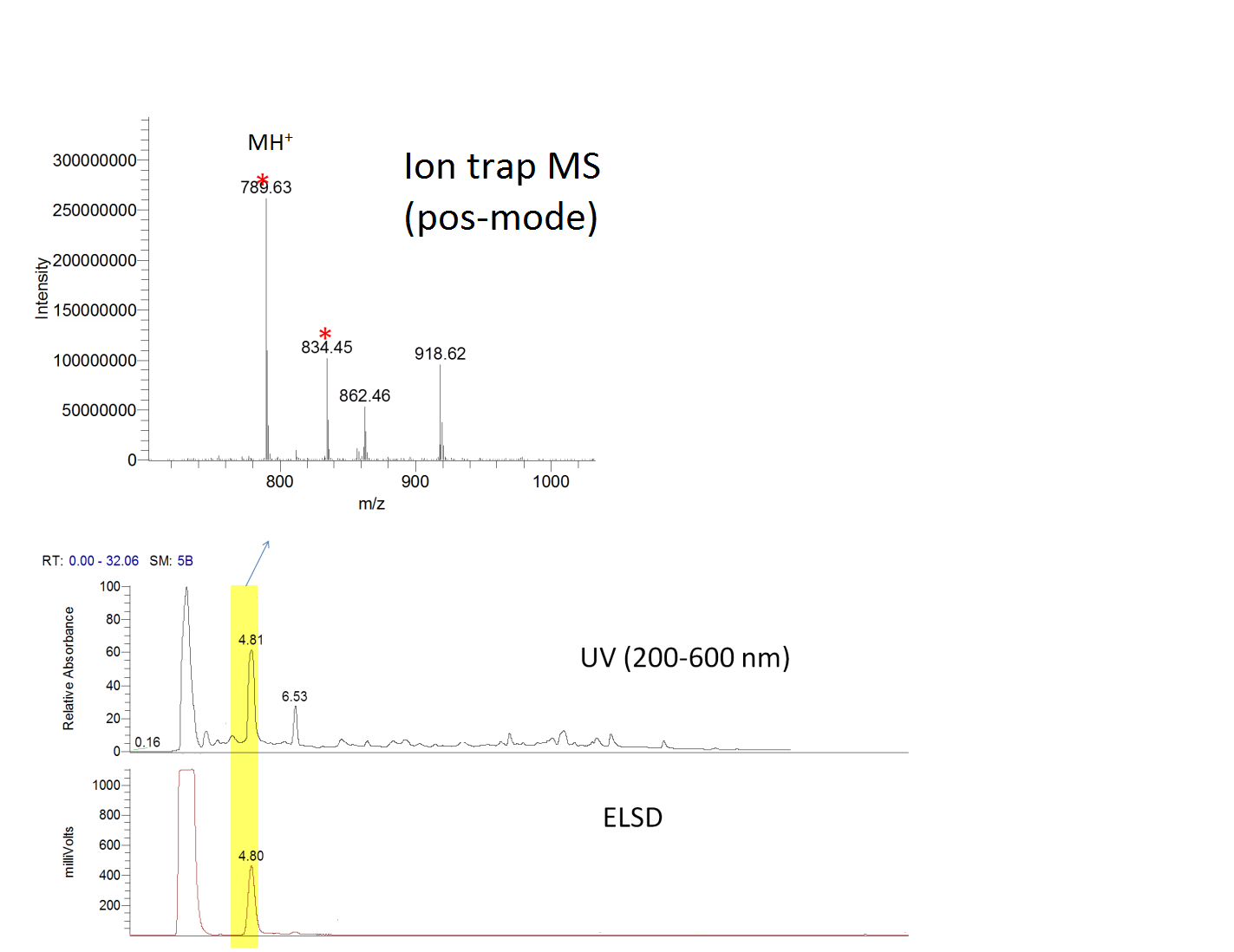


**Figure S5.** LCMS analysis of active fraction A3 in fraction plate FP1. The molecular weight of the major component was determined as 788.6 ± 0.2 Da based on monomer and dimer adducts, which guided the isolation of nuiapolide (**1**).


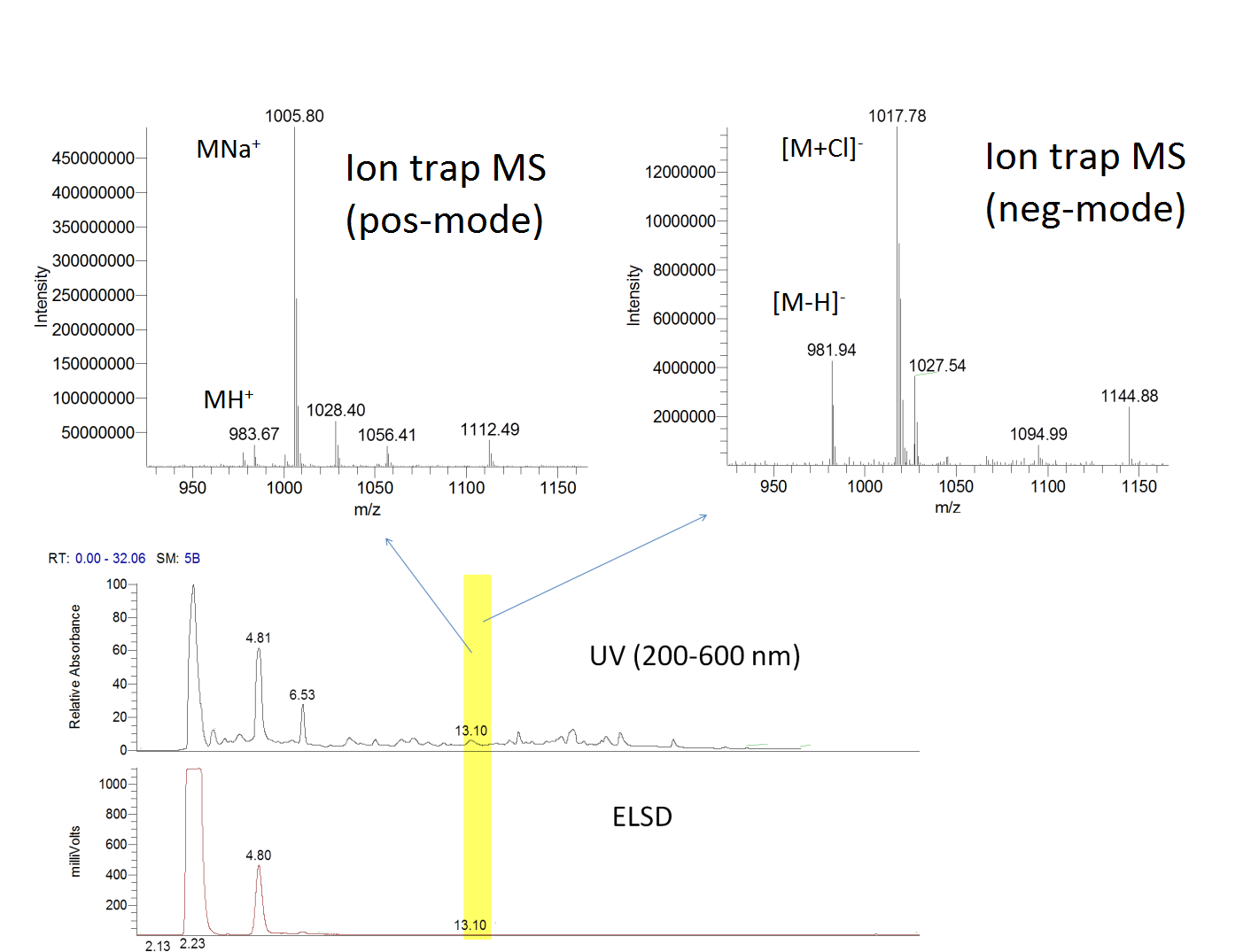


**Figure S6.** LCMS analysis of active fractions A11/12 in fraction plate FP1. The molecular weight of the major component was determined as 982.8 ± 0.2 Da based on monomer adducts, which guided isolation of NP982.

| 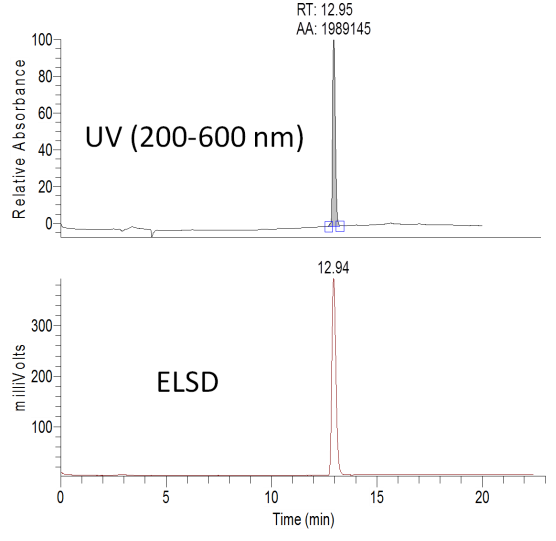 |
| --- |
| (**a**) |
| 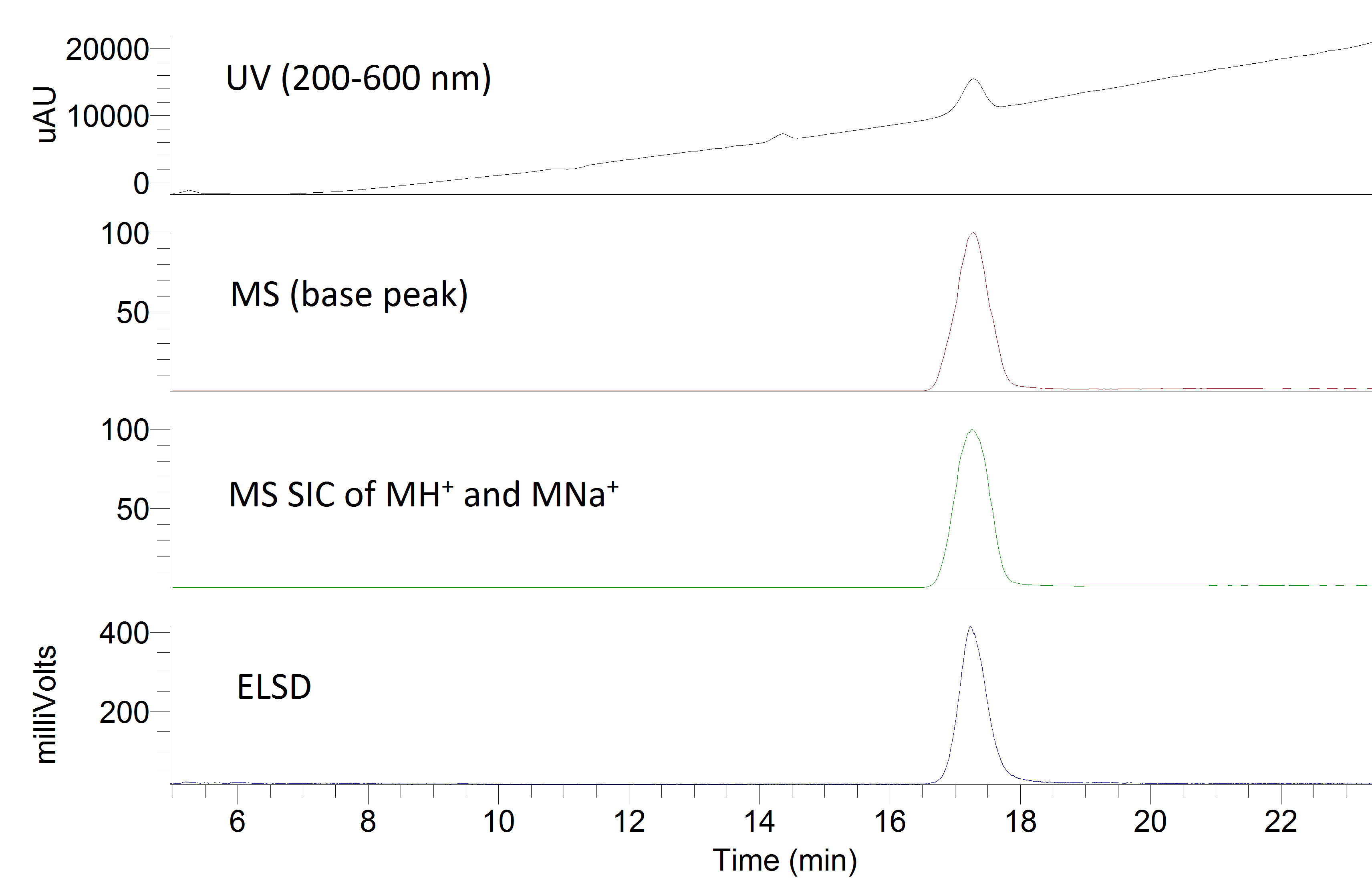 |
| (**b**) |

**Figure S7.** Purified (**a**) nuiapolide (**1**) and (**b**) NP982. Nuiapolide (**1**) was purified in sufficient quantities for structural analysis. NP982 was purified but low yields and multiple conformations precluded complete analysis.


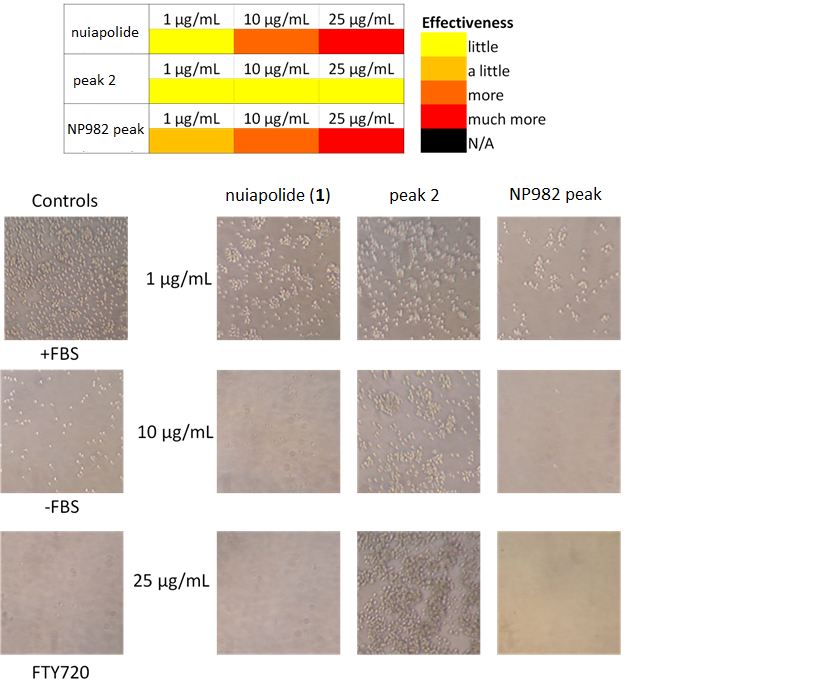


**Figure S8.** Chemotaxis assay of FP1 active fractions: The fractions, including purified nuiapolide (**1**) and an NP982-containing fraction, were assessed for the ability to inhibit chemotaxis of Jurkat cells at concentrations of 1, 10, and 25 µg/mL. Peak 2 eluted between the two active fractions (see Figure S2a, peak at 6.5 min) and showed no activity.


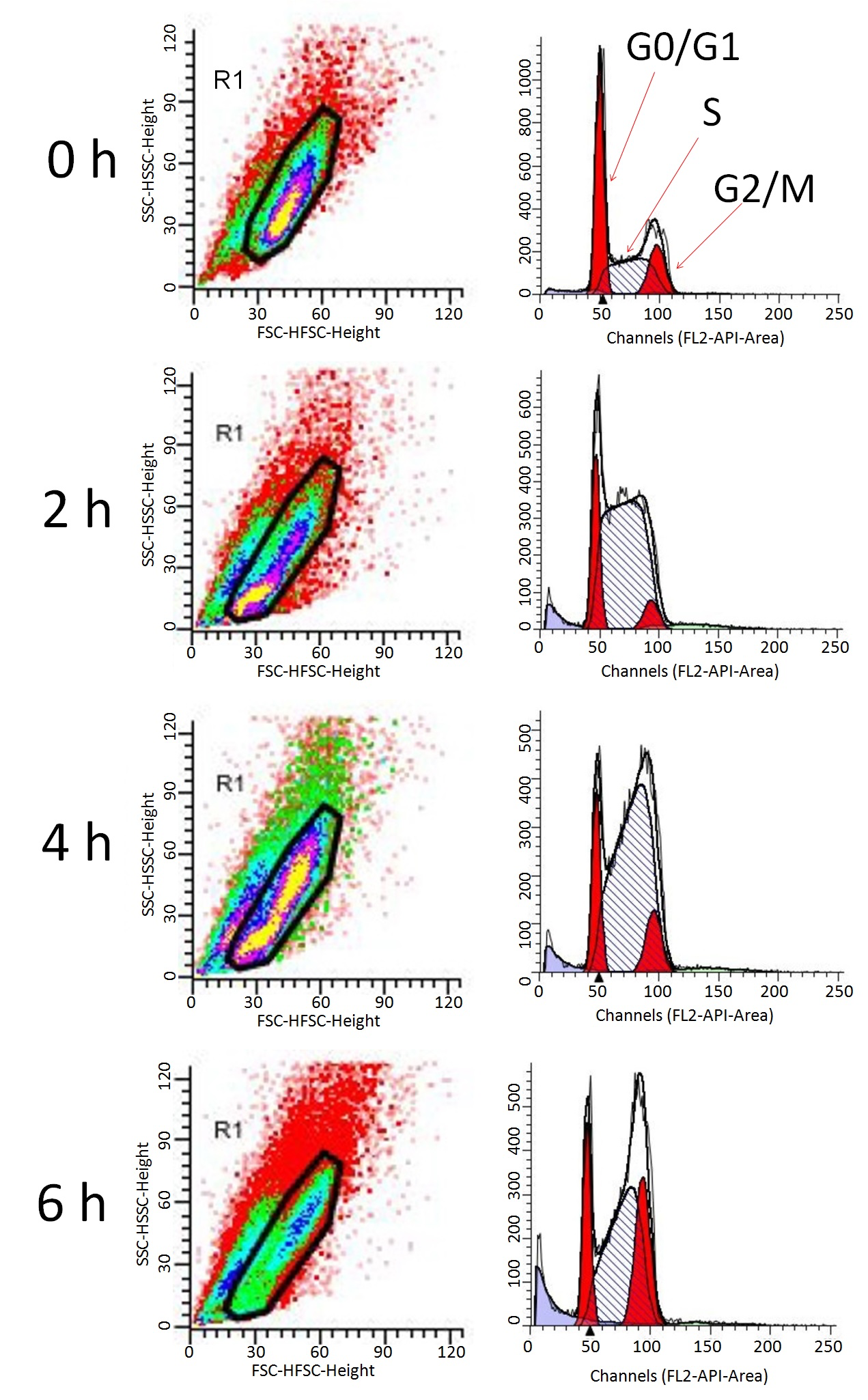


**Figure S9.** Cell cycle analysis of nuiapolide (**1**) treated Jurkat cells. Left: light-scattering properties of cells/nuclei and Right: Histogram with modeled cell cycle components.


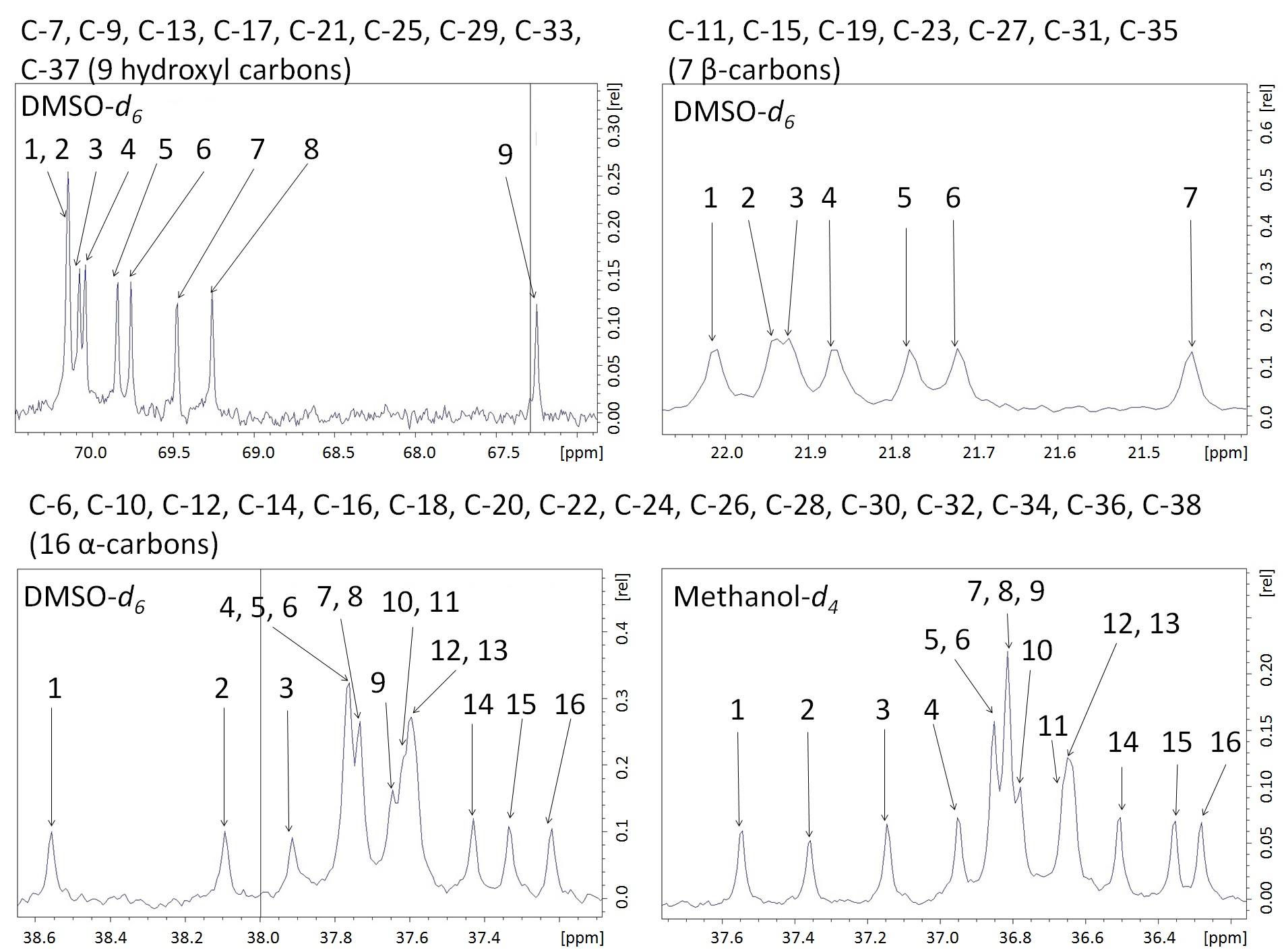


**Figure S10.** Number of carbons in ^13^C NMR grouped regions. Top right: 21.4–22.0 ppm in DMSO-*d*_6_. Top left: 69.2–70.2 ppm in DMSO-*d*_6_. Bottom right: 36.2–37.4 ppm in methanol-*d*_4_. Bottom left: 37.2–38.1 ppm in DMSO-*d*_6_.

Spectral Data of Nuaipolide (1)

Nuaipolide (**1**): ^1^H NMR (methanol-*d*_4_, 500 MHz): δ_H_ 0.93 (9H, s, CH_3_41, CH_3_42, and CH_3_43),
1.31–1.55 (14H, overlapped m, CH_2_11, CH_2_15, CH_2_19, CH_2_23, CH_2_27, CH_2_31, and CH_2_35), 1.48–1.59 (26H, overlapped m, CH_2_10, CH_2_12, CH_2_14, CH_2_16, CH_2_18, CH_2_20, CH_2_22, CH_2_24, CH_2_26, CH_2_28, CH_2_30, CH_2_32, and CH_2_34), 1.42 (2H, m, CH_2_36), 1.49 (2H, m, CH_2_6), 1.56 (2H, m, CH_2_8), 1.58 (2H, m, CH_2_38), 1.60 (2H, m, CH_2_5), 1.96 (3H, s, CH_3_44), 2.58 (1H, m, H4′), 2.83 (1H, m, H4″), 3.42 (1H, m, H37), 3.57 (6H, s, H13, H17, H21, H25, H29, and H33), 3.78 (2H, s, H7 and H9), 5.02 (1H, dd,
*J* = 9.7, *J* = 2.5 Hz, H39) 5.74 (1H, s, H2). ^1^H NMR (dimethyl sulfoxide-*d*_6_, 500 MHz): δ_H_ 0.85 (9H, s, CH_3_41, CH_3_42, and CH_3_43), 1.19–1.37 (14H, overlapped m, CH_2_11, CH_2_15, CH_2_19, CH_2_23, CH_2_27, CH_2_31, and CH_2_35), 1.24–1.41 (26H, overlapped m, CH_2_10, CH_2_12, CH_2_14, CH_2_16, CH_2_18, CH_2_20, CH_2_22, CH_2_24, CH_2_26, CH_2_28, CH_2_30, CH_2_32, and CH_2_34), 1.28 (2H, m, CH_2_36), 1.31 (2H, m, CH_2_6), 1.37 (2H, m, CH_2_8), 1.44 (2H, m, CH_2_38), 1.49 (2H, m, CH_2_5), 1.87 (3H, s, CH_3_44), 2.59 (2H, m, CH_2_4), 3.25 (1H, m, H37), 3.37 (6H, s, H13, H17, H21, H25, H29, and H33), 3.58 (2H, s, H7 and H9), 4.14 (6H, d, *J* = 4.1 Hz, OH13, OH17, OH21, OH25, OH29, and OH33), 4.15 (1H, d, *J* = 5.6 Hz, OH37), 4.44 (1H, d, *J* = 4.4 Hz, OH7 or OH9), 4.47 (1H, d, *J* = 4.2 Hz, OH7 or OH9), 4.94 (1H, dd,
*J* = 9.7, *J* = 2.5 Hz, H39), 5.67 (1H, s, H2). ^13^C NMR (methanol-*d*_4_, 500 MHz): δ_C_ 20.8–21.5 (C11, C15, C19, C23, C27, C31, and C35), 23.86 (C5), 23.89 (C44), 25.00 (C41, C42, and C43), 32.69 (C4), 33.91 (C40), 36.2–37.4 (C6, C10, C12, C14, C16, C18, C20, C22, C24, C26, C28, C30, C32, C34, and C36), 37.55 (C38), 43.51 (C8), 67.58 (C37), 69.8–70.8 (C7, C9, C13, C17, C21, C25, C29, and C33), 76.82 (C39), 115.73 (C2), 161.20 (C3), 166.90 (C1). ^13^C NMR (dimethyl sulfoxide-*d*_6_, 500 MHz):
δ_C_ 21.4–22.0 (C11, C15, C19, C23, C27, C31, and C35), 24.08 (C5), 24.91 (C44), 26.27 (C41, C42, and C43), 33.11 (C4), 34.60 (C40), 37.2–38.1 (C6, C10, C12, C14, C16, C18, C20, C22, C24, C26, C28, C30, C32, C34, and C36), 38.56 (C38), 44.67 (C8), 67.25 (C37), 69.2–70.2 (C7, C9, C13, C17, C21, C25, C29, and C33), 76.91 (C39), 116.69 (C2), 160.04 (C3), 166.04 (C1). HR-TOF-ESIMS,
*m*/*z* [M + H]^+^ 789.6076 (calcd for C_44_H_85_O_11_, 789.6092, Δ −2.2 ppm), [M + Na]^+^ 811.5895 (calcd for C_44_H_84_O_11_Na, 811.5911, Δ −2.0 ppm).


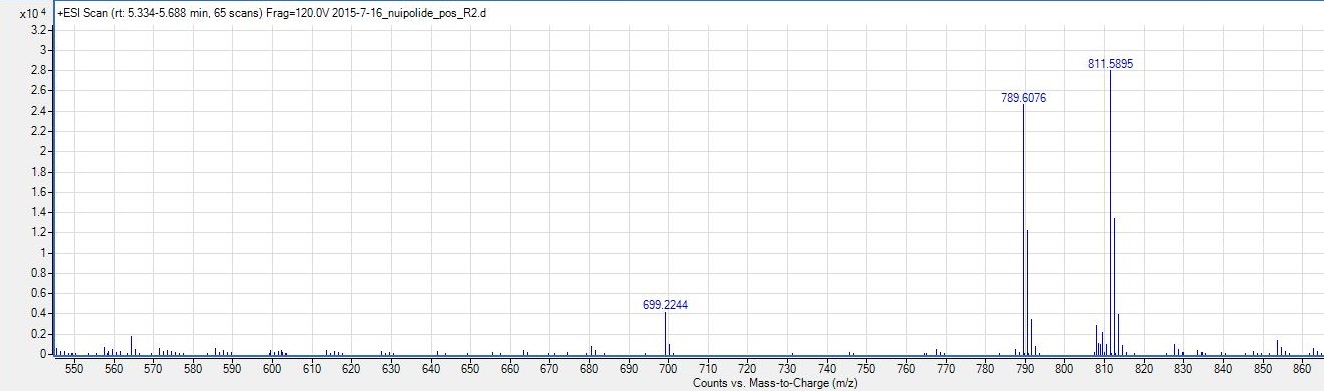


**Figure S11.** Spectral data of nuiapolide (**1**).


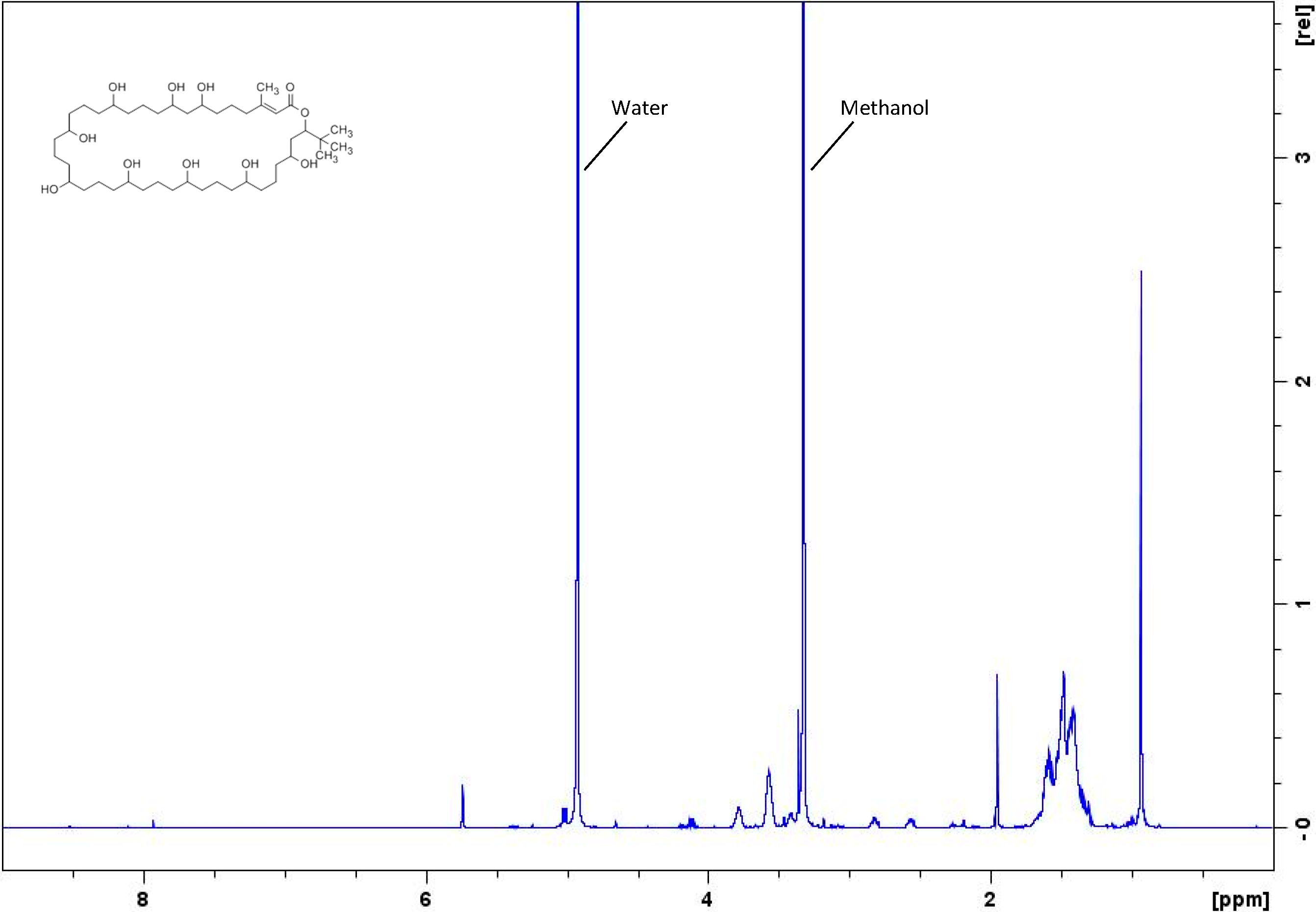


**Figure S12.** ^1^H NMR of Nuiapolide (**1**) in Methanol-*d*_4_.


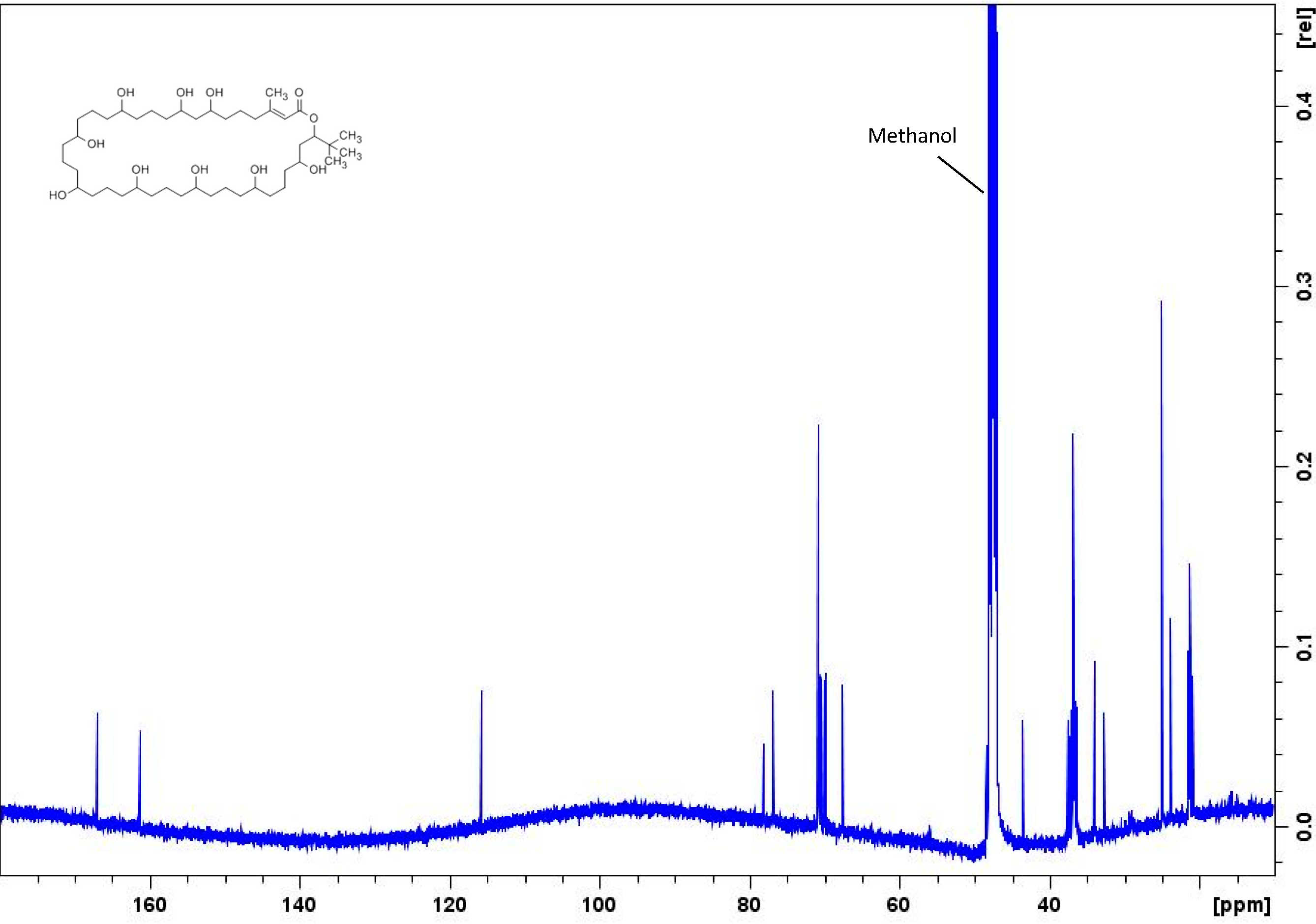


**Figure S13.** ^13^C NMR of Nuiapolide (**1**) in Methanol-*d*_4_.


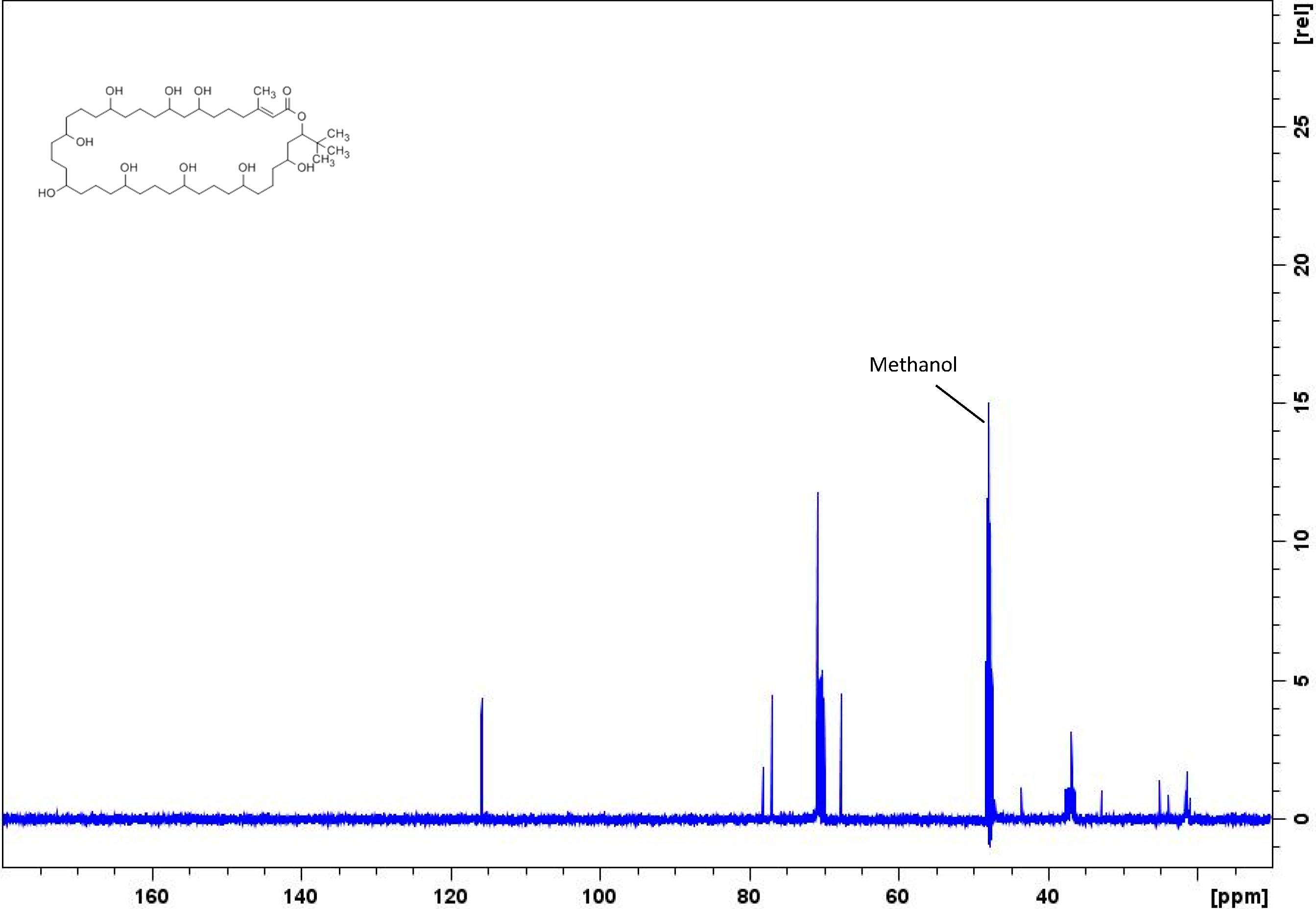


**Figure S14.** DEPT-90 of Nuiapolide (**1**) in Methanol-*d*_4_.


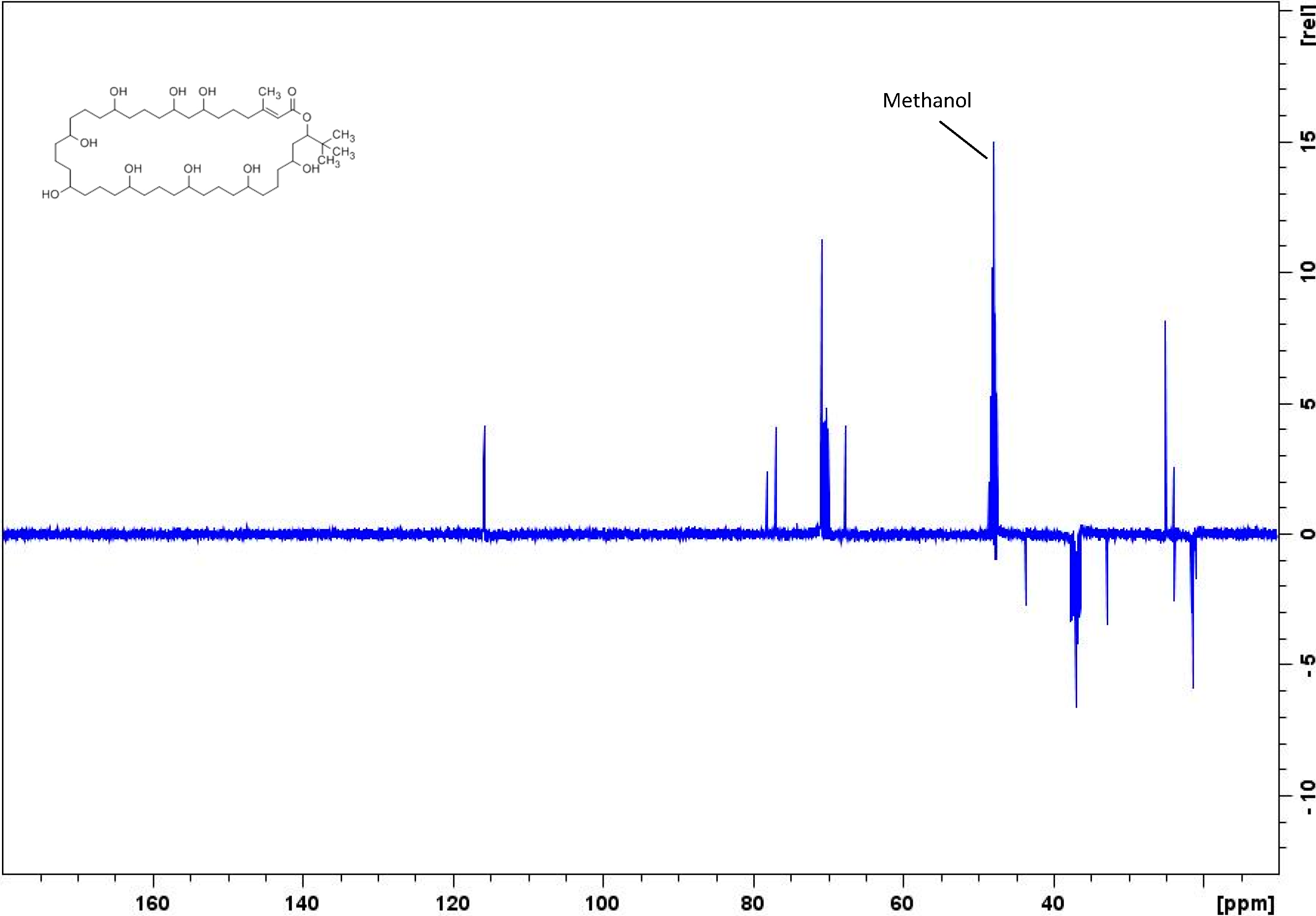


**Figure S15.** DEPT-135 of Nuiapolide (**1**) in Methanol-*d*_4_.

**Figure S16.** COSY of Nuiapolide (**1**) in Methanol-*d*_4_.


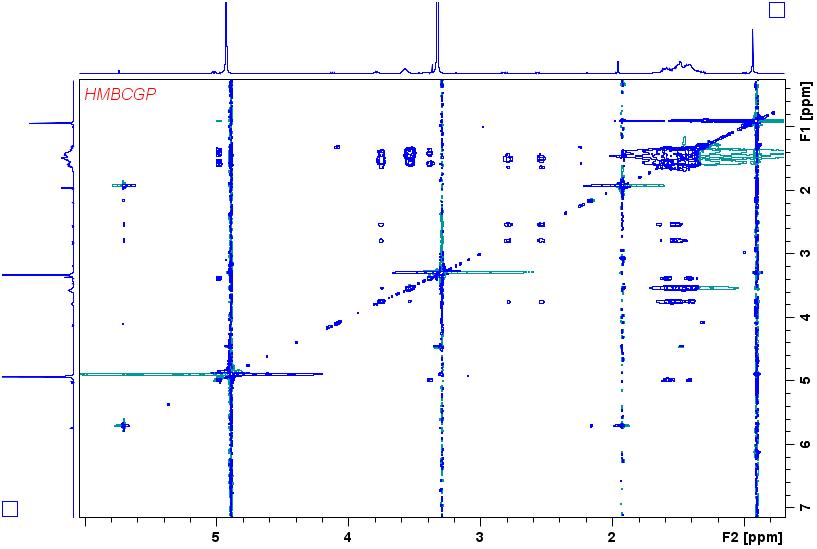


**Figure S17.** TCOSY of Nuiapolide (**1**) in Methanol-*d*_4_.


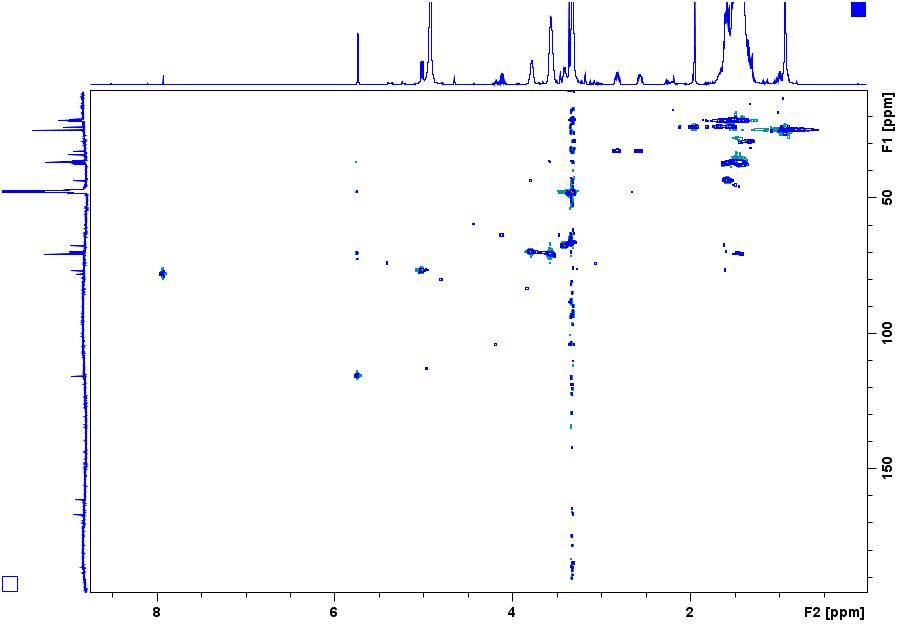


**Figure S18.** HSQC of Nuiapolide (**1**) in Methanol-*d*_4_.


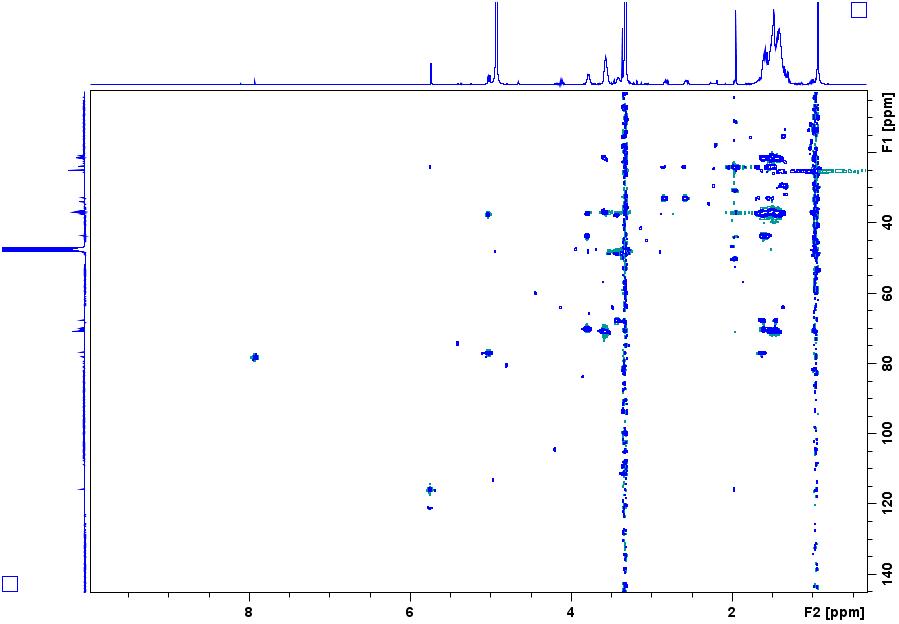


**Figure S19.** HSQC-TOCSY of Nuiapolide (**1**) in Methanol-*d*_4_.


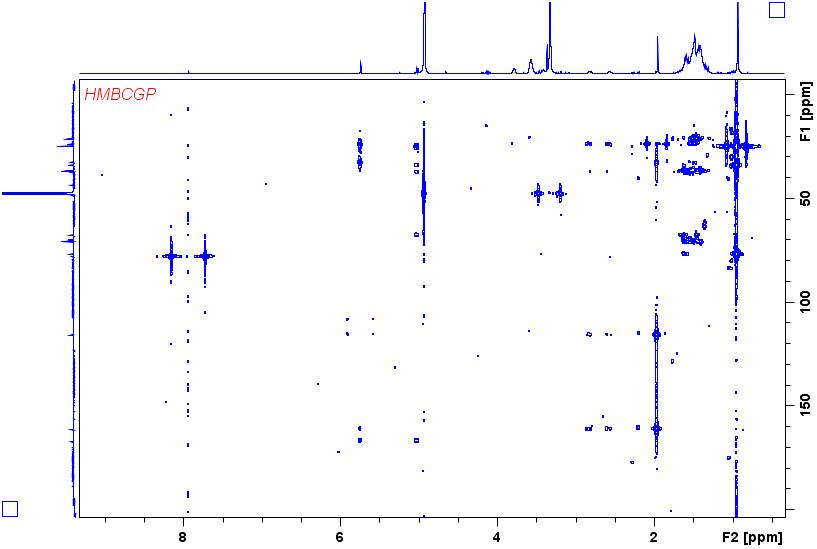


**Figure S20.** HMBC of Nuiapolide (**1**) in Methanol-*d*_4_.


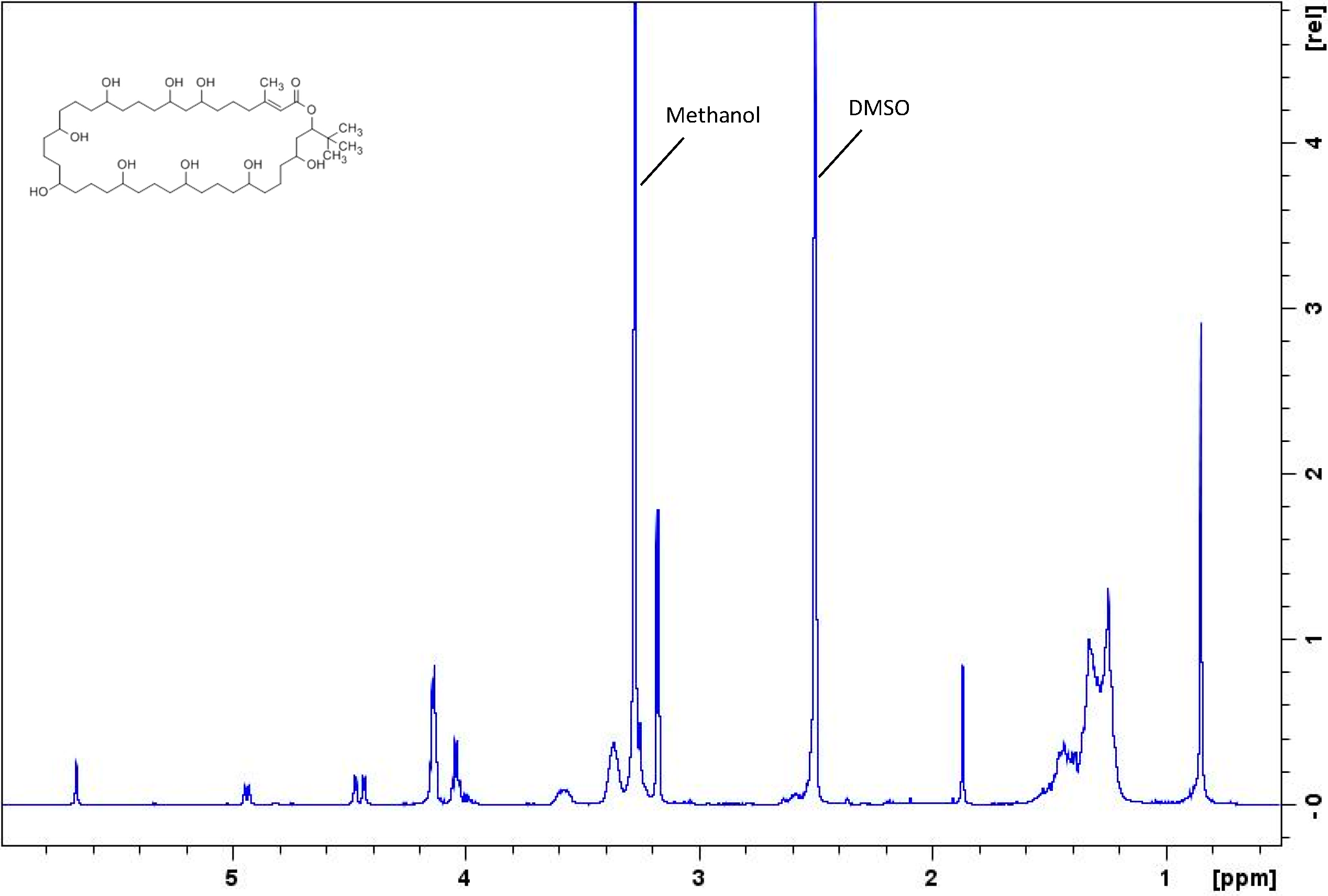


**Figure S21.** ^1^H NMR of Nuiapolide (**1**) in DMSO-*d*_6_.


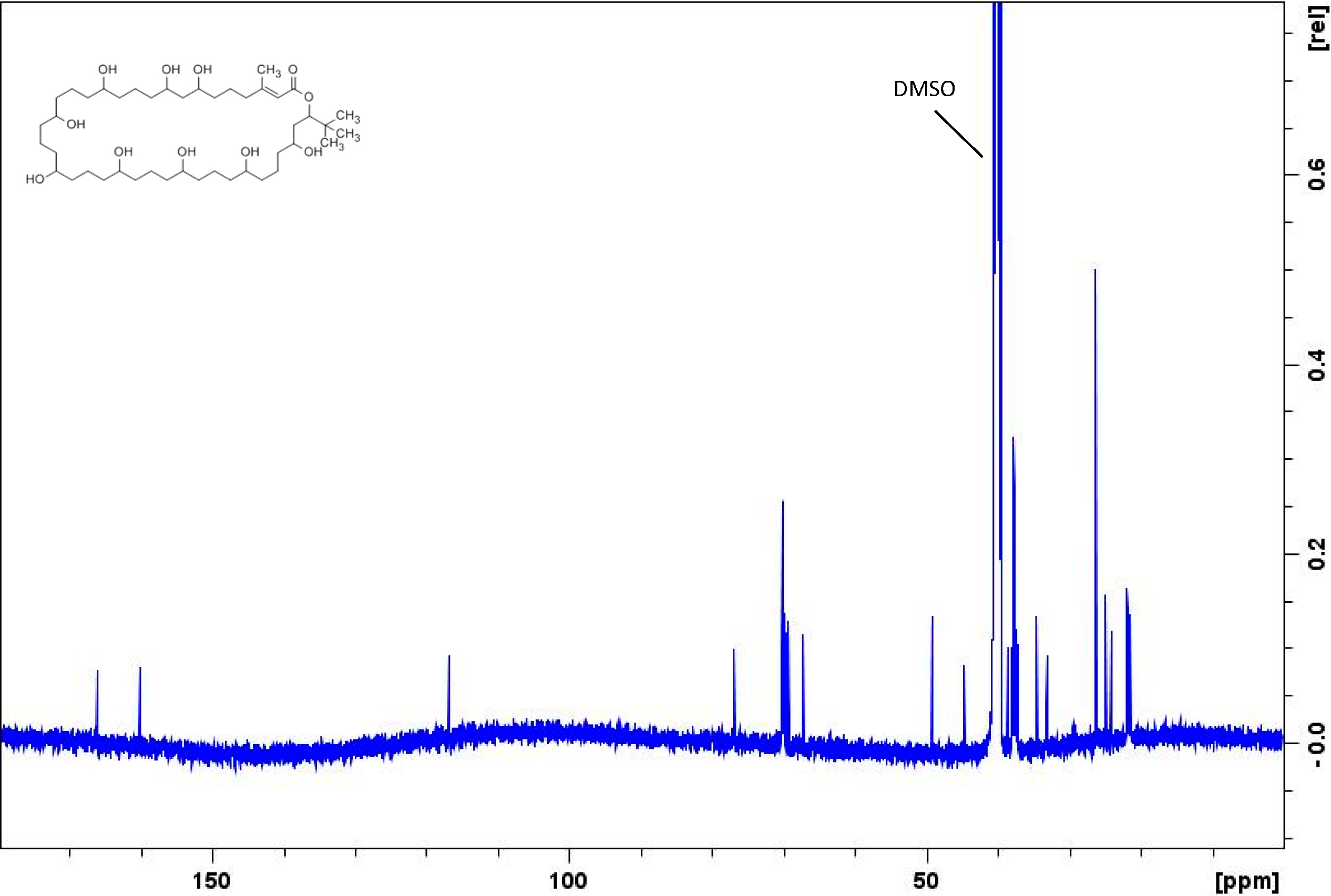


**Figure S22.** ^13^C NMR of Nuiapolide (**1**) in DMSO-*d*_6_.


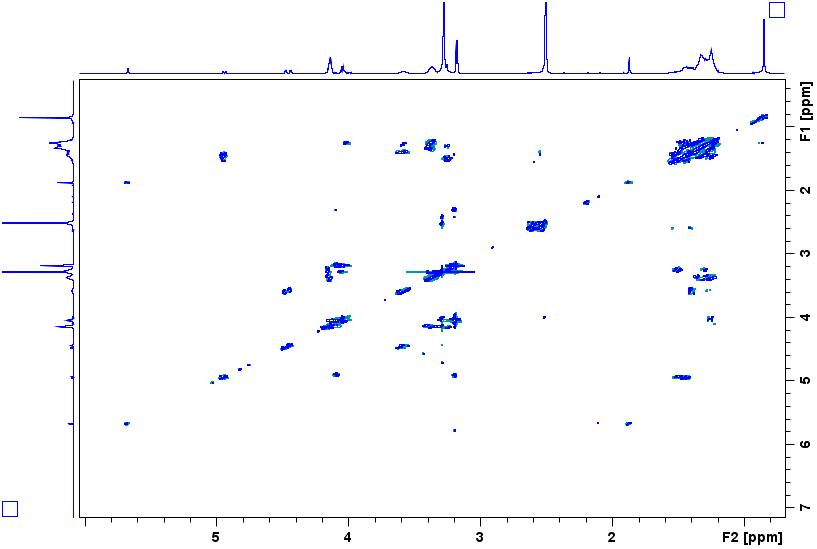


**Figure S23.** COSY of Nuiapolide (**1**) in DMSO-*d*_6_.


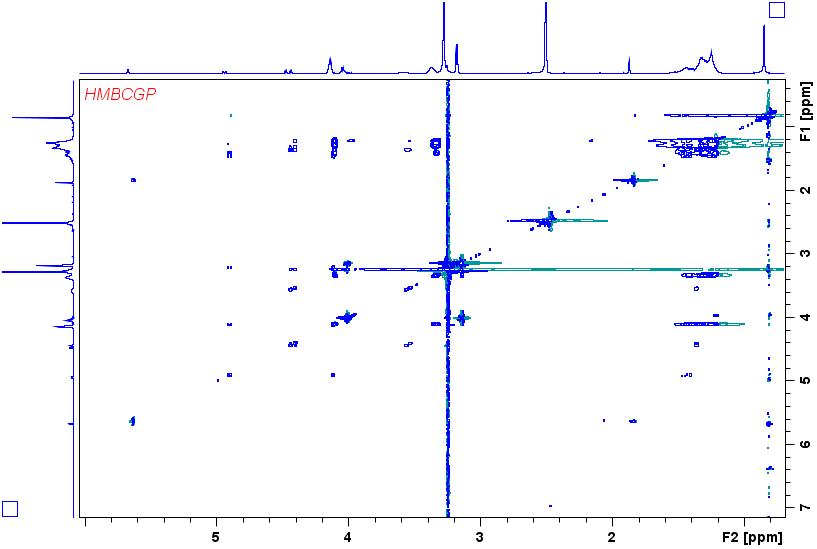


**Figure S24.** TCOSY of Nuiapolide (**1**) in DMSO-*d*_6_.


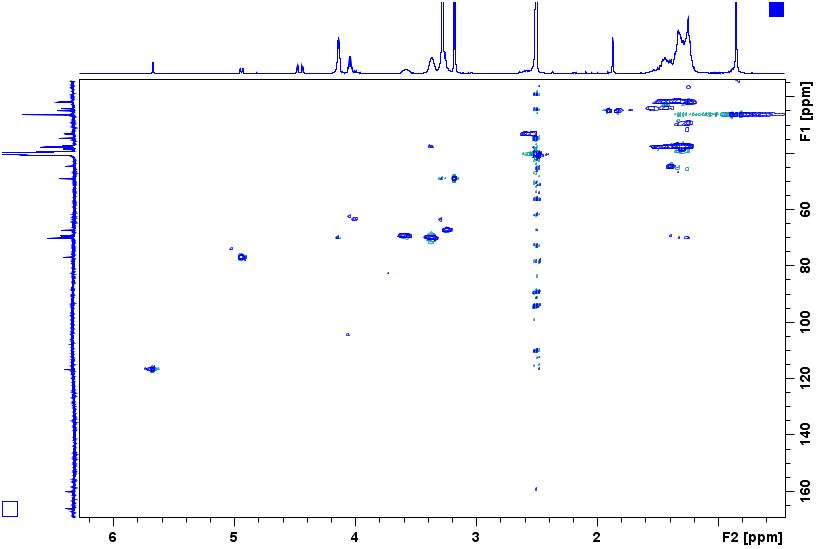


**Figure S25.** HSQC of Nuiapolide (**1**) in DMSO-*d*_6_.

**Figure S26.** HMBC of Nuiapolide (**1**) in DMSO-*d*_6_.

© 2015 by the authors; licensee MDPI, Basel, Switzerland. This article is an open access article distributed under the terms and conditions of the Creative Commons Attribution license (http://creativecommons.org/licenses/by/4.0/).
